# Synthesis of Ti_4_Au_3_C_3_ and its derivative trilayer goldene through chemical exfoliation

**DOI:** 10.1126/sciadv.adt7999

**Published:** 2025-03-28

**Authors:** Yuchen Shi, Shun Kashiwaya, Jun Lu, Martin Dahlqvist, Davide G. Sangiovanni, Vladyslav Rogoz, Martin Magnuson, Grzegorz Greczynski, Mike Andersson, Johanna Rosen, Lars Hultman

**Affiliations:** ^1^ Materials Design Division, Department of Physics, Chemistry and Biology (IFM), Linköping University, SE-581 83 Linköping, Sweden.; ^2^ Thin Film Physics Division, Department of Physics, Chemistry and Biology (IFM), Linköping University, SE-581 83 Linköping, Sweden.; ^3^ Theoretical Physics Division, Department of Physics, Chemistry and Biology (IFM), Linköping University, SE-581 83 Linköping, Sweden.; ^4^ Division of Sensor and Actuator Systems, Department of Physics, Chemistry and Biology (IFM), Linköping University, SE-581 83 Linköping, Sweden.; ^5^ Wallenberg Initiative Materials Science for Sustainability, Department of Physics, Chemistry, and Biology (IFM), Linköping University, SE-581 83 Linköping, Sweden.; ^6^ Center for Plasma and Thin Film Technologies, Ming Chi University of Technology, New Taipei City 24301, Taiwan.

## Abstract

Achieving large two-dimensional (2D) sheets of any metal is challenging due to their tendency to coalescence or cluster into 3D shapes. Recently, single-atom-thick gold sheets, termed goldene, was reported. Here, we ask if goldene can be extended to include multiple layers. The answer is yes, and trilayer goldene is the magic number, for reasons of electronegativity. Experiments are made to synthesize the atomically laminated phase Ti_4_Au_3_C_3_ through substitutional intercalation of Si layers in Ti_4_SiC_3_ for Au. Density functional theory calculations suggest that it is energetically favorable to insert three layers of Au into Ti_4_SiC_3_, compared to inserting a monolayer, a bilayer, or more than three layers. Isolated trilayer goldene sheets, ~100 nanometers wide and 6.7 angstroms thick, were obtained by chemically etching the Ti_4_C_3_ layers from Ti_4_Au_3_C_3_ templates. Furthermore, trilayer goldene is found in both *hcp* and *fcc* forms, where the *hcp* is ~50 milli–electron volts per atom more stable at room temperature from ab initio molecular dynamic simulations.

## Teaser

Goldene takes on more forms, where trilayer is the magic number.

## INTRODUCTION

Single-atom-thick sheets of gold, termed goldene, was recently discovered (*1*). Beyond the well-explored realm of graphene, a rich landscape of two-dimensional (2D) materials emerges, each with distinctive characteristics and applications (*2*, *3*). Among these materials, gold—renowned for its noble nature and catalytic prowess—has demonstrated notable potential in the form of nanoparticles for practical applications in biosensing, catalysis, and gene therapy (*4*–*6*). When confined to an atomically thin layer, gold exhibits fascinating properties, further broadening its potential in various fields. Bandgap opening and spin-orbit splitting have been reported in substrate-supported monolayer Au (*7*, *8*), qualifying it for optoelectronic and spintronic devices. Because of the high surface area–to–volume ratio, 2D configurations offer more active sites that can participate in electrochemical reactions, thereby enhancing the catalytic performance (*9*). The interaction of 2D Au with light, owing to the quantum confinement effect, results in intensified surface plasmon resonance, which plays a key role in maximizing solar light absorption and scattering (*10*). Density functional theory (DFT) calculations predict that 1D Au nanorods and 2D Au nanoprisms with thiolate-ligand passivation could improve near-infrared absorption properties (*11*).

Nevertheless, 2D materials are often susceptible to structural instabilities due to their high surface area. Gold typically forms 3D metallic bonds, and forcing it into a 2D configuration can lead to structural instability and reconstruction. Therefore, achieving precise control over the size, shape, thickness, and quality of 2D materials is crucial for progress. Efforts have been made to fabricate atomically thin Au membranes through physical or chemical methods. [001]-Oriented Au nanosheets with a thickness of 0.2 to 0.4 nm (1 to 2 atomic layers) were synthesized and stabilized within the confined space provided by layered double hydroxides (*12*). Wang *et al*. (*13*) fabricated alleged free-standing monoatomic thick Au by dealloying bulk Au-Ag crystals through electron beam irradiation. Free-standing two-atomic-thick Au nanosheets were synthesized via a wet-chemical route using methyl orange as a surfactant, which exhibited very high catalytic activity (*9*, *14*). It has been reported that free-standing monolayer Au with nanoribbon structures can suspend in graphene pores (*15*). Monolayer gold quantum dots were coated onto hexagonal boron nitride (BN) surfaces using pulsed laser deposition, demonstrating tunable bandgaps depending on their size and shape (*7*). Au was thermally intercalated and stabilized as 2D configuration with a single atomic thickness between silicon carbide and monolayer graphene (*8*). In addition, single-layer Au can be intercalated between graphene layers to form a graphene-goldene-graphene structure with weak interlayer interactions (*16*). Microwave synthesis of atomically thin Au crystals and their 2D hybrids with graphene, BN, and molybdenum disulfide (MoS_2_) has also been demonstrated (*17*). Theoretically, DFT and ab initio molecular dynamics (AIMD) have predicted stable free-standing 2D monolayer of Au (*1*, *18*, *19*). First-principles calculations suggested that goldene has excellent conductivity at room temperature, potentially reaching the same order of magnitudes as carrier-doped graphene (*20*). The protocol for preparing single-atom-layer goldene (*1*) involves etching away the M and X layers (T_3_C_2_ sheets) from the Ti_3_AuC_2_ MAX phase using an alkaline potassium ferricyanide solution (Murakami’s reagent) together with surfactants, leaving free-standing single-atom-thick Au layers. M_*n*+1_AX*_n_* phases are a class of materials that combines metallic and ceramic properties (*21*). The acronym “MAX” stands for the three main constituents of these compounds: M represents an early transition metal, A is an element from group IIIA or IVA of the periodic table, and X denotes carbon or nitrogen. The layers consist of transition metal carbide or nitride slabs interleaved with A-group elements. Selective etching of the A layers produces 2D M_*n*+1_X*_n_* sheets, known as MXenes, with the most well-known example being Ti_3_C_2_ (*22*, *23*).

The question now arises: Can goldene be generalized to a varying number of atomic layers in a series 1, 2, 3, …? The study of multilayer goldene would help understand how additional layers influence physical and chemical properties of 2D layered materials and provide a test platform for studying quantum confinement and interlayer coupling effects. Trilayer goldene, depending on how its atomic layers are stacked, could support the exploration of exotic electronic states of noble metals. A recent study (*24*) indicated the possibility to make percolation-free quasi-2D Au films (thickness down to 3.5 nm) using graphene-inspired technology, providing inspiration for goldene exfoliation.

Here, we report isolated 2D trilayer-thick Au sheets (termed trilayer goldene), with a thickness of 6.7 Å, achieved by etching away Ti_4_C_3_ from Ti_4_Au_3_C_3_. The template, Ti_4_Au_3_C_3_, is a rising MAX phase formed by thermal annealing of Au-capped Ti_4_SiC_3_ thin films, where Si is completely replaced by Au. The trilayer goldene can adopt both *ABA* and *ABC* stackings (*hcp* and *fcc* structures) due to similar thermodynamic free energy and dynamic stability, as confirmed by DFT calculations and molecular dynamic simulations. The Ti_4_AuC_3_ phase is also found to be attainable, but it is energetically less stable than Ti_4_Au_3_C_3_ with respect to competing compound formation. Furthermore, the electronic properties of the trilayer goldene were studied by x-ray photoelectron spectroscopy (XPS). A family of goldene allotropes is thus discovered.

## RESULTS

### Synthesis of Ti_4_Au_3_C_3_

**Fig. 1.**
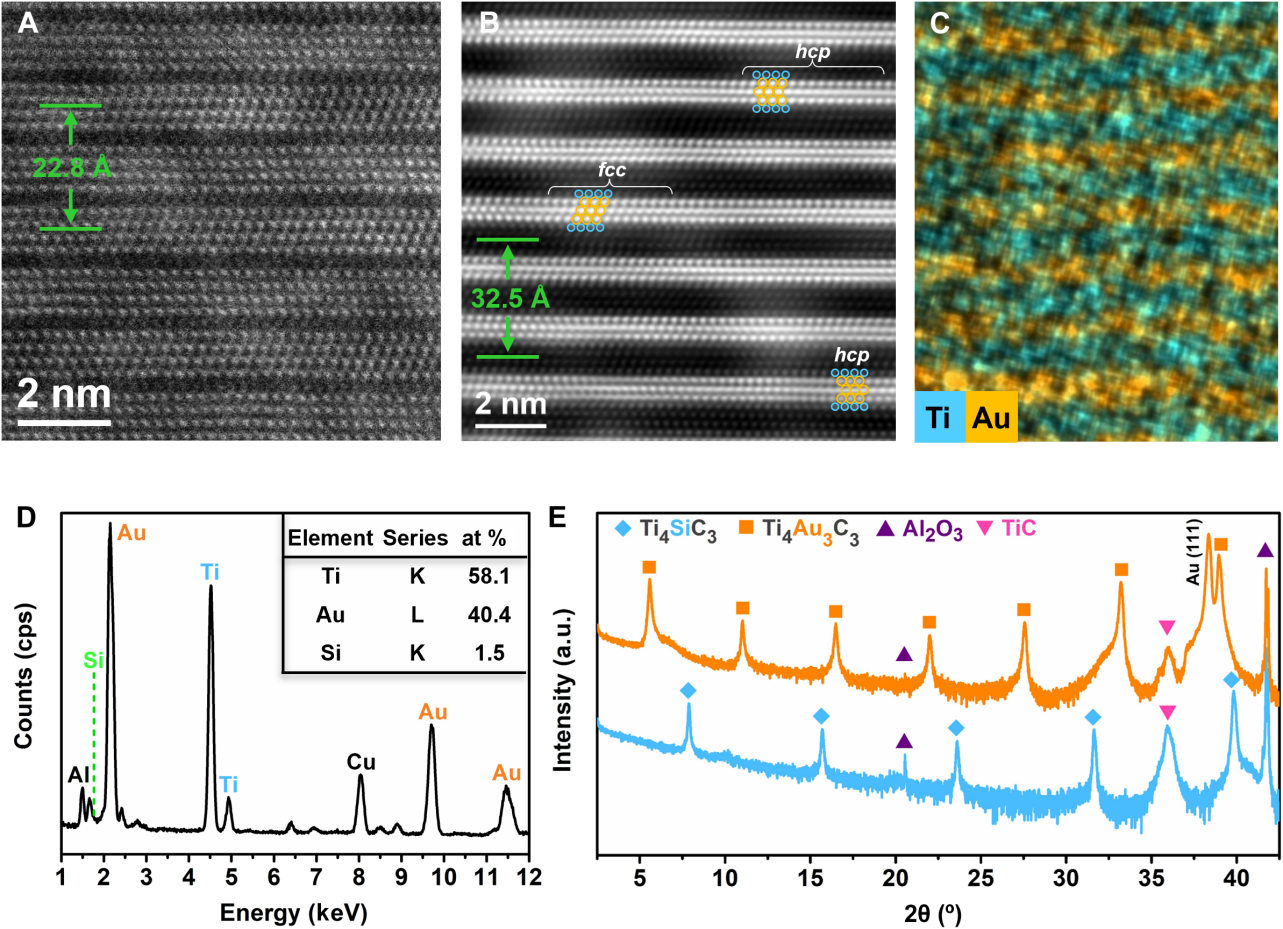
Synthesis of Ti_4_Au_3_C_3_. (**A**) HRSTEM image of sputter-deposited Ti_4_SiC_3_. (**B**) HRSTEM image of the Ti_4_Au_3_C_3_ phase obtained after 30 hours of annealing at 670°C. Both STEM images were recorded along the [$11\bar{2}0$] direction. Ti and Au atoms are circled in light blue and yellow, respectively. (**C**) Corresponding EDX elemental mapping of (B). (**D**) EDX spectrum of Ti_4_Au_3_C_3_ phase showing atomic ratio of Ti, Au, and C elements. (**E**) XRD patterns of Ti_4_SiC_3_ and Au-covered Ti_4_SiC_3_ after 30 hours of annealing at 670°C. at %, atomic %; cps, counts per second; a.u., arbitrary units.

Figure 1A shows a high-resolution scanning transmission electron microscopy (HRSTEM) image of a sputter-deposited epitaxial Ti_4_SiC_3_ film on Al_2_O_3_(0001) substrates with a TiC(111) seed layer, taken along the [$11\bar{2}0$] direction. The image reveals a characteristic laminated structure where Ti_4_C_3_ sheets are sandwiched between Si (c.f. silicene) layers. The film was sputter-coated with a 200-nm-thick Au layer, and the Au-covered Ti_4_SiC_3_ sample was then annealed at 670°C for 30 hours in a N_2_ atmosphere to obtain a high-quality Ti-Au-C phase. The HRSTEM image and correspoding energy-dispersive x-ray (EDX) map of the annealed sample in Fig. 1 (B and C) show a characteristic nanolaminated structure corresponding to the M_4_A_3_X_3_ phase, where Ti_4_C_3_ sheets are now sandwiched between three-atom-thick Au layers. The *c*-lattice parameter increased from 22.8 Å for Ti_4_SiC_3_ to 32.5 Å for Ti_4_Au_3_C_3_, reflecting a 42.5% increase. Despite this conspicuous lattice expansion, the coherent laminate structure is retained. The expansion is attributed to the insertion of Au, where each Au layer consists of atoms larger than those of the original Si in the A layer. The Ti and Au signals in the EDX map align well with the alternating laminated Ti and Au layers, confirming the insertion and location of Au. The corresponding EDX spectrum shows the presence of Ti, Au, and Si (Fig. 1D). The relative atomic ratio of Ti:(Au + Si) is about 4:3, consistent with the stoichiometry of a hybrid 433 MAX phase, as shown in the inset table of Fig. 1D. The low content of Si (at the 1.5 atomic % detection level) indicates that the Au intercalation was nearly complete. Figure 1E shows the x-ray diffraction (XRD) patterns of the as-grown Ti_4_SiC_3_ film and the annealed Au-covered Ti_4_SiC_3_ sample. The corresponding (000*l*) peaks shifted to lower angles after annealing, reflecting the lattice expansion along the *c* axis. The *c* parameter is calculated to be ~32.3 Å from the (0004) peak, which is in good agreement with the value obtained from direct observation in the scanning transmission electron microscopy (STEM) image (Fig. 1B).

The mechanism of the substitution intercalation reaction can be explained by the loosely bonded Si atoms that are provided with a reduced chemical potential path to diffuse out into the Au capping layer, leaving behind vacancies that are subsequently backfilled with Au. This process is facilitated by the interdiffusion abilities of the two elements, in conjunction with their eutectic phase diagram (*25*, *26*). Detailed mechanism can be found in section S1. Similar transformations of Ti_3_Au*_x_*C_2_ (*x* = 1 and 2) from Ti_3_SiC_2_ and Ti_*n*+1_Au_2_C*_n_* from Ti_*n*+1_AlC*_n_* (*n* = 1 and 2) have been reported (*25*, *27*). An attractive aspect of this work is the demonstration that three atomic layers of Au can substitute each Si layer in Ti_4_SiC_3_.

In large-scale STEM images (section S1), peculiar examples of intercalation frontlines for the trilayer Au were observed, where the second and third gold layers closely follow the first layer, effectively pushing apart two adjacent Ti_4_C_3_ sheets. The deformation of the Ti_4_C_3_ sheets is conspicuous during the insertion of three atomic layers of Au, as indicated by the blue lines. Ti_3_C_2_T*_x_* sheets have shown a comparable Young’s modulus and exhibit high ductility, tensile strength, as well as toughness (*22*, *28*). Similarly, high elasticity is expected for Ti_4_C_3_ sheets, which explains why the laminated structure was retained despite undergoing notable deformation and lattice expansion.

**Fig. 2.**
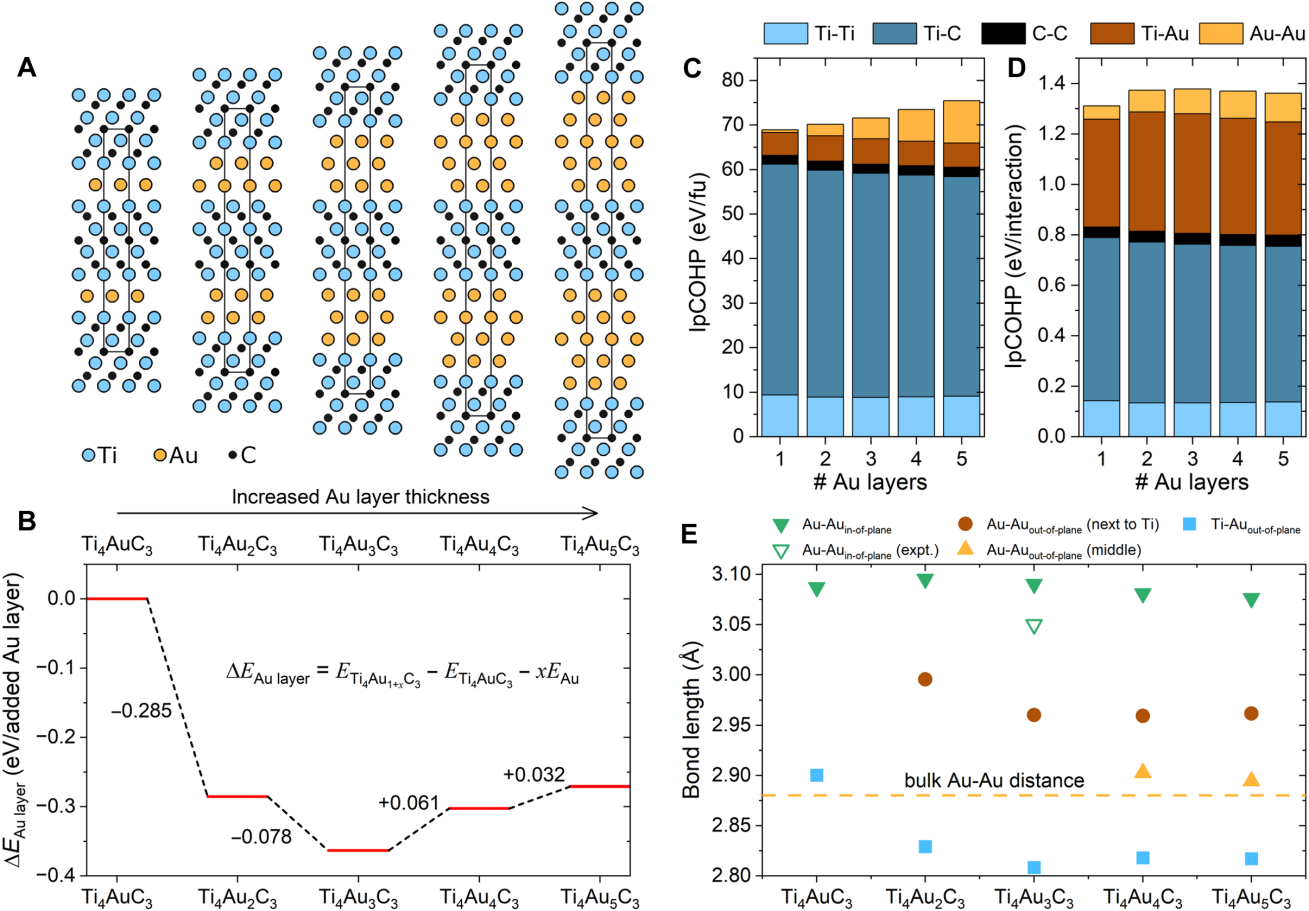
DFT calculations for Ti_4_Au_1+***x***_C_3_ phases. (**A**) Schematic illustration of increasing number of Au layers in Ti_4_Au_1+*x*_C_3_ with Ti, Au, and C atoms colored in blue, gold, and black, respectively. (**B**) Change in energy for Ti_4_Au_1+*x*_C_3_ upon inserting layers of Au to Ti_4_AuC_3_. Bonding analysis in terms of integrated partial crystal orbital Hamiltonian population (IpCOHP) given (**C**) per formula unit (fu) and (**D**) per interaction. (**E**) Bond lengths for structures depicted in (A).

The intercalation of monolayer Au in Ti_4_SiC_3_ was also explored at lower annealing temperatures with shorter durations to synthesize Ti_4_AuC_3_ (section S2); however, this phase is not prevalent. To investigate the impact of inserting additional layers of Au into Ti_4_AuC_3_ from a theoretical perspective, multiple structures were considered. These are illustrated in section S3 and include different stackings of the Au layers as well as different stackings of the Ti_4_C_3_ subunits relative to the Au layers. Their energies have been calculed using DFT, with a focus on the configurations found to be lowest in energy. These are shown in Fig. 2A with calculated lattice parameters and space group symmetries provided in table S1. It is important to note that for Ti_4_Au_3_C_3_, Ti_4_Au_4_C_3_, and Ti_4_Au_5_C_3_, there are additional structures with alternative stacking of Au that are close in energy to those illustrated in Fig. 2A. The common feature of all these low-energy structures is that the Au layers are stacked in an *fcc*, *hcp*, or mixed configuration. Using eq. S1, we calculated the energy gain or cost associated with inserting additional layers into Ti_4_AuC_3_. Figure 2B shows that it is energetically favorable to insert additional layers of Au into Ti_4_AuC_3_, as indicated by the negative energy change of −0.285 eV when transitioning from Ti_4_AuC_3_ to Ti_4_Au_2_C_3_ and −0.078 eV when transitioning from Ti_4_Au_2_C_3_ to Ti_4_Au_3_C_3_. At three layers of Au, i.e., Ti_4_Au_3_C_3_, a minimum energy of −0.363 eV per inserted Au layer is achieved relative to Ti_4_AuC_3_. The addition of a fourth or fifth Au layer, however, will cost energy by +0.061 eV and +0.032 eV, respectively, when transitioning from Ti_4_Au_3_C_3_ to Ti_4_Au_4_C_3_ and Ti_4_Au_5_C_3_. Additional phase stability data for Ti_4_Au_1+*x*_C_3_ compared to its set of most competing phases can be found in table S2.

A reason why three layers of gold (Ti_4_Au_3_C_3_) are energetically most favorable, as shown in Fig. 2B, is revealed through the bonding analysis in Fig. 2 (C and D) and fig. S11, combined with the evaluation of bond lengths shown in Fig. 2E. For Ti_4_AuC_3_, Au-Au interactions are only found in-plane. However, when additional layers of Au are inserted into the structure, out-of-plane Au-Au interactions appear. Beyond the trilayer Au configuration, for the Au layer in the middle, out-of-plane Au-Au interactions occur without direct bonding to Ti. This positively affects bonding, as seen in Fig. 2C, where the integrated partial crystal orbital Hamiltonian population increases with additional Au layers. Analysis of the individual contributions in Fig. 2D shows that Ti-Au interactions also benefit from the presence of multiple Au layers. Figure 2D further demonstrates that three layers of Au in Ti_4_Au_3_C_3_ provide the largest total individual contribution. This is corroborated by the bond lengths shown in Fig. 2E, where Ti-Au and Au-Au bonds (adjacent to the Ti layer) are shortest for Ti_4_Au_3_C_3_, indicating improved bonding strengths compared to configurations with fewer—or more—than three Au layers. The experimentally calibrated in-plane Au-Au distances, which are slightly shorter than the calculated values for Ti_4_Au_3_C_3_ embedded in Fig. 2E, are found in section S4.

Another perspective on the relative stability of the trilayer goldene is from the difference in electronegativity between Au (2.54), Ti (1.54), and C (2.55) compared to Si (1.90) or Al (1.61). As Au is more electronegative than Ti, there will be electronic charge transfer from Ti to Au. The stability of goldene stacks would then be a delicate balance of charge transfer for the metal bonding between the Au atoms and a trilayer of Au happens to cause the “right” amount of charge transfer from Ti to Au for its highest stability, compared to less or more gold layers (see section S4, including test DFT calculations).

### Atomic stackings in trilayers Au in Ti_4_Au_3_C_3_

In this study, we found that trilayers of Au in Ti_4_Au_3_C_3_ contain both *ABA* and *ABC* stacking configurations (Fig. 1B and section S4), corresponding to 2H *hcp* and *fcc* structures, respectively. The coexistence of *hcp* and *fcc* stacking does not lead to a spread in the *c* parameter. For the *fcc* phase of Au, the calculated *a* parameter is 3.091 Å and the *c* parameter is 32.529 Å. These values are very similar to those of the *hcp* phase of Au (table S1), which aligns with the experimental observations (section S4). The Ti_4_Au_3_C_3_ phase with *ABA* stacking of Au has an energy of −0.363 eV, whereas the *ABC* stacking has an energy of −0.354 eV (fig. S8). Both trilayer structures are therefore feasible as stable Au allotropes. From simulated *ABA* structures (fig. S8), Ti_4_C_3_ sheets are mirrored with Au layers. In contrast, for the *ABC* stacking, Ti_4_C_3_ sheets stack in a zig-zag pattern along [$11\bar{2}0$] with respect to the Au layers. Since the Ti_4_C_3_ sheets in Ti_4_SiC_3_ and Ti_4_AuC_3_ are mirrored with A layers (Si or Au layers), as observations in STEM images and simulated structures (Fig. 1A and figs. S2 and S6), the position of the Ti_4_C_3_ sheets did not shift laterally when the monolayer Au was introduced. This also applies to the Ti_4_Au_3_C_3_ with *ABA*-stacked Au, where the Ti_4_C_3_ sheets did not shift. To achieve the formation of *ABC*-stacked Au layers, displacement of two adjacent Ti_4_C_3_ sheets is required. Such a displacement was observed in STEM images (Fig. 1B), where Ti atoms move laterally by approximately 0.5 Å relative to their original mirrored positions. Similar phenomena have also been observed when inserting bilayer Au into Ti_3_SiC_2_ to form Ti_3_Au_2_C_2_ (*25*). However, gliding of M_*n*+1_X*_n_* slabs in the MAX family has not previously been reported. A possible mechanism for Ti_4_C_3_ gliding is discussed in section S5.

The coexistence of *hcp* and *fcc* quasi-goldene sheets can be understood by considering the reduced dimensionality of the metal, which increases the proportion of surface energy relative to the total system energy. Consequently, material properties can be tuned through crystal phase engineering. For instance, *hcp* metals often exhibit anisotropic properties due to the directional arrangement of atoms, while *fcc* metals typically have isotropic properties. These anisotropic materials are of interest across various disciplines, because of unique properties. Compared to their *fcc* counterparts, *hcp* Au nanoparticles have shown plasmon and interband transitions (*29*), and a 130-fold increase in in-plane resistivity and more pronounced plasmon absorption have been demonstrated in 4H Ag (*30*). Zhang’s group (*31*, *32*) synthesized ~2.4-nm-thick 2H *hcp* Au nanosquare sheets and 4H Au nanoribbons using wet-chemical methods. Ye *et al*. (*9*) reported an *hcp* Au phase existing at the edge of two-atomic-thick *fcc* Au nanosheets. Kondo and Takayanagi (*33*) synthesized *fcc* Au nanowires encapsuled by an *hcp* Au outer shell. Such non-*fcc* crystalline phase is known to stabilize ultrathin Au nanostructures (*34*, *35*). An *hcp*/*fcc* alternating structure was observed in a square-like Au plate due to the *hcp* to *fcc* transformation as the plate grows thicker (*36*). To the best of our knowledge, the finding of large-scale, isolated, subnanometer-thick Au sheets with *hcp* structures is also original.

### Preparation of trilayer goldene

**Fig. 3.**
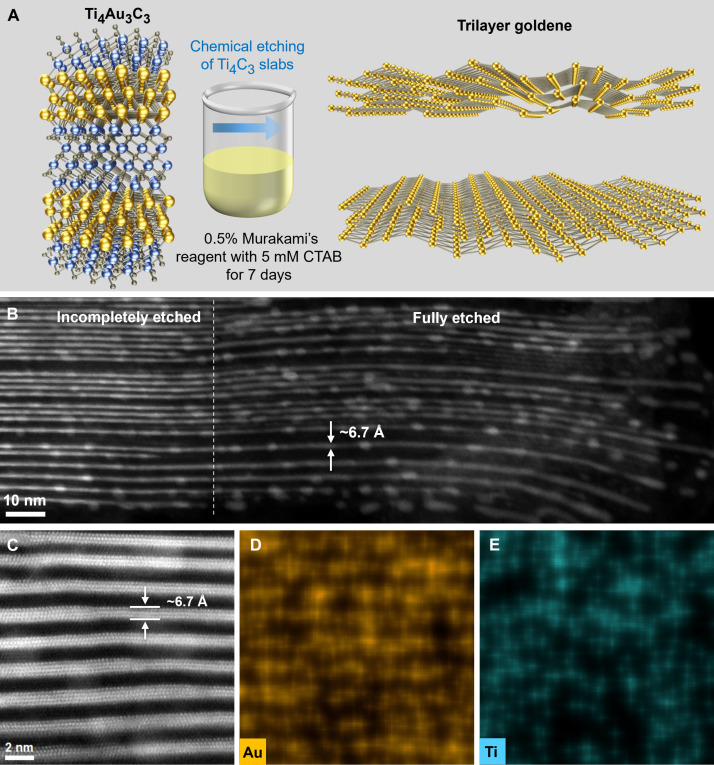
Preparation of trilayer goldene. (**A**) Schematic illustration of the trilayer goldene preparation. (**B**) Cross-sectional HRSTEM image of an etching frontline, where the original Ti_4_Au_3_C_3_ structure remains at the left side and isolated trilayer goldene sheets appear at the right side. (**C**) A magnified HRSTEM image of trilayer goldene extracted from fig. S19 and its corresponding EDX elemental mapping of Au (**D**) and Ti (**E**).

To obtain isolated trilayer goldene, Ti_4_C_3_ sheets were selectively etched away using 0.5% Murakami’s reagent with 5 mM cetrimonium bromide (CTAB) for 168 hours. A schematic illustration is shown in Fig. 3A. Ti_4_C_3_ sheets are stepwise oxidized by radical nascent oxygen [O] generated in an alkaline solution of potassium ferricyanide (K_3_[Fe(CN)_6_] in KOH) (*1*, *37*). CTAB surfactant was used to permeate the gaps once Ti_4_C_3_ slabs between freed and to impede the agglomeration of 2D gold layers into multilayers or nanoparticles, as applied for gold nanoparticles (*38*, *39*). We obtained an average in-plane Au-Au spacing of 2.86 Å in trilayer goldene (section S6), which is close to the equilibrium interatomic distance in *fcc* bulk Au (2.884 Å) and slightly larger than the DFT-calculated values (section S7). The Au-Au spacing in trilayer goldene is approximately 6.5% smaller than that in Ti_4_Au_3_C_3_ due to lattice contraction after exfoliation. Figure 3B shows an etching frontline observed at the edge of a Ti_4_Au_3_C_3_ film, where trilayer goldene is being exfoliated from the right side. The goldene sheets remain separate after etching but begin to ripple from the edges once losing the support of the Ti_4_C_3_ sheets. The gap opening observed at the edges can be attributed to the fact that a CTAB chain, with a length of up to 20 Å, is larger than the distance between goldene sheets. CTAB molecules, which bind vertically to Au surfaces in a bilayer formation (*40*), can expand the gaps between adjacent goldene sheets. The goldene sheets in the more deeply etched regions (the central area of the image) appear to maintain relatively better flatness, likely due to a slower infiltration of CTAB molecules parallel to them. Initial results indicate that an etched-free trilayer goldene is more stable than the corresponding monolayers (*1*) and can maintain its structure with ripple features extending over a hundred nanometers. Meanwhile, blob formation of Au at the edges and their lateral diffusion through the sheets occur (fig. S19). A magnified image reveals some four- and five-layer goldene sheets near the edges, as shown in Fig. 3C. The formation of blobs and thicker layers can be attributed to the rapid interactions between the released goldene sheets and excess spurious Au atoms during etching processes (*1*). In addition, this phenomenon is related to the coalescence of goldene sheets during ion milling in the transmission electron microscopy sample preparation processes (fig. S20). The black regions in Fig. 3C clearly indicate the complete removal of Ti_4_C_3_ sheets and confirm that the thickness of the three-atomic-layer Au is approximately 6.7 Å. The thickness of trilayer goldene inside of Ti_4_Au_3_C_3_ is measured to be 6.7 Å (see Fig. 3B). The corresponding EDX map of Au exhibits a distinct lamellar signal, while Ti is evenly dispersed (Fig. 3, D and E), further confirming the complete etching. Solutions with higher concentration of Murakami’s reagent would be more aggressive toward Ti_4_Au_3_C_3_, leading to faster etching of Ti_4_C_3_ sheets and the formation of Au particles through sheet clustering and curling up of sheets. Conversely, etching with a lower concentration of 0.2% requires a much longer time to achieve complete etching (fig. S21). Results about etching without CTAB and the stability of trilayer goldene can be found in figs. S22 and S23. In addition, AIMD simulations have shown that two adjacent goldene layers that contain Si impurities coalesce in few picoseconds if their interlayer spacing is within 7 Å (*1*). The goldene-goldene interaction weakens substantially as the interlayer spacing increases from 10 to 12 Å and 14 Å. In this work, the distance between Au sheets is about 12 Å in Ti_4_Au_3_C_3_, which is larger than the ~9.3-Å spacing in Ti_3_AuC_2_. In contrast, the coalescence of two adjacent trilayer goldene after etching was less observed, as wider channels are provided for surfactants to stabilize the free-standing goldene sheets. Furthermore, the as-synthesized Ti_4_Au_3_C_3_ film is very close to ideal stoichiometry with negligible Si content, as confirmed by STEM-EDX and XRD (Fig. 1), implying that any disturbance of goldene layers by Si impurities is minimal.

Pure Au *hcp* square sheets have been found to become stable under ambient conditions when they are less than ~6 nm thick (*31*), and ultrathin nanowires have been stabilized by *hcp* surface structures (*33*). Therefore, it is not unexpected that the present trilayer goldene contains *hcp*-stacked regions. These factors collectively suggest promising potential for the production of larger quasi-2D Au sheets. The process yield of trilayer goldene is discussed in section S6.

### DFT and AIMD investigations of free-standing trilayer goldene

To investigate the dynamic and energetic stability of *fcc*-like and *hcp*-like trilayer goldene sheets, DFT calcuations were carried out at 0 K and AIMD simulations at 300 K (section S7). DFT calculations show that *ABA* and *ABC* trilayer Au slabs have nearly equal energy. For both Perdew-Burke-Ernzerhof and local density approximation results, the energy difference between *fcc* and *hcp* slabs is less than 1 meV/atom. It has been experimentaly demonstrated that the *hcp* phase exists in Au nanostructures (*41*), as the stacking fault energy in *fcc* metals is quite low.

On-the-fly machine learning–assisted AIMD simulations at 300 K were conducted to verify the dynamical stability of *fcc-* and *hcp*-stacked trilayer goldene. The dynamics of the trilayers were monitored for 0.18 ns (*fcc*) and 0.38 ns (*hcp*). The simulations indicate that both *fcc* and *hcp* stackings are dynamically stable, as shown by time-averaged Au positions in fig. S25. After a brief transient period, during which the Au layers shift to their equilibrium separation distance, the atoms continue vibrating around their respective *fcc* (or *hcp*) lattice positions for the entire simulation. The time-averaged potential energies suggest that, at room temperature, the *hcp* stacking is approximately 45 meV/atom more stable than the *fcc* stacking. An additional contribution arises from the vibrational free energy (*F*_vib_), with the *hcp*-structured sheet being further stabilized by 5 meV/atom more than the *fcc* (fig. S24). Accordingly, the Helmholtz free energy (*F*) of the two allotropes, obtained by adding *F*_vib_ to the time-averaged potential energies, shows a difference of ∆*F*_*hcp*-*fcc*_ ≈ 50 meV/atom, indicating that *hcp* trilayer Au is substantially more stable than *fcc* trilayer Au at 300 K. Therefore, we predict that both structures would be retained after etching, with the *hcp* structure likely being more prevalent, as inferenced from the dominant *ABA*-Au in Ti_4_Au_3_C_3_ (section S4). *hcp*-structured trilayer goldene is observed (fig. S18). The prevalence of trilayer goldene may thus be attributed to the relative stability of the *hcp* stacking.

### Electronic properties of trilayer goldene

**Fig. 4.**
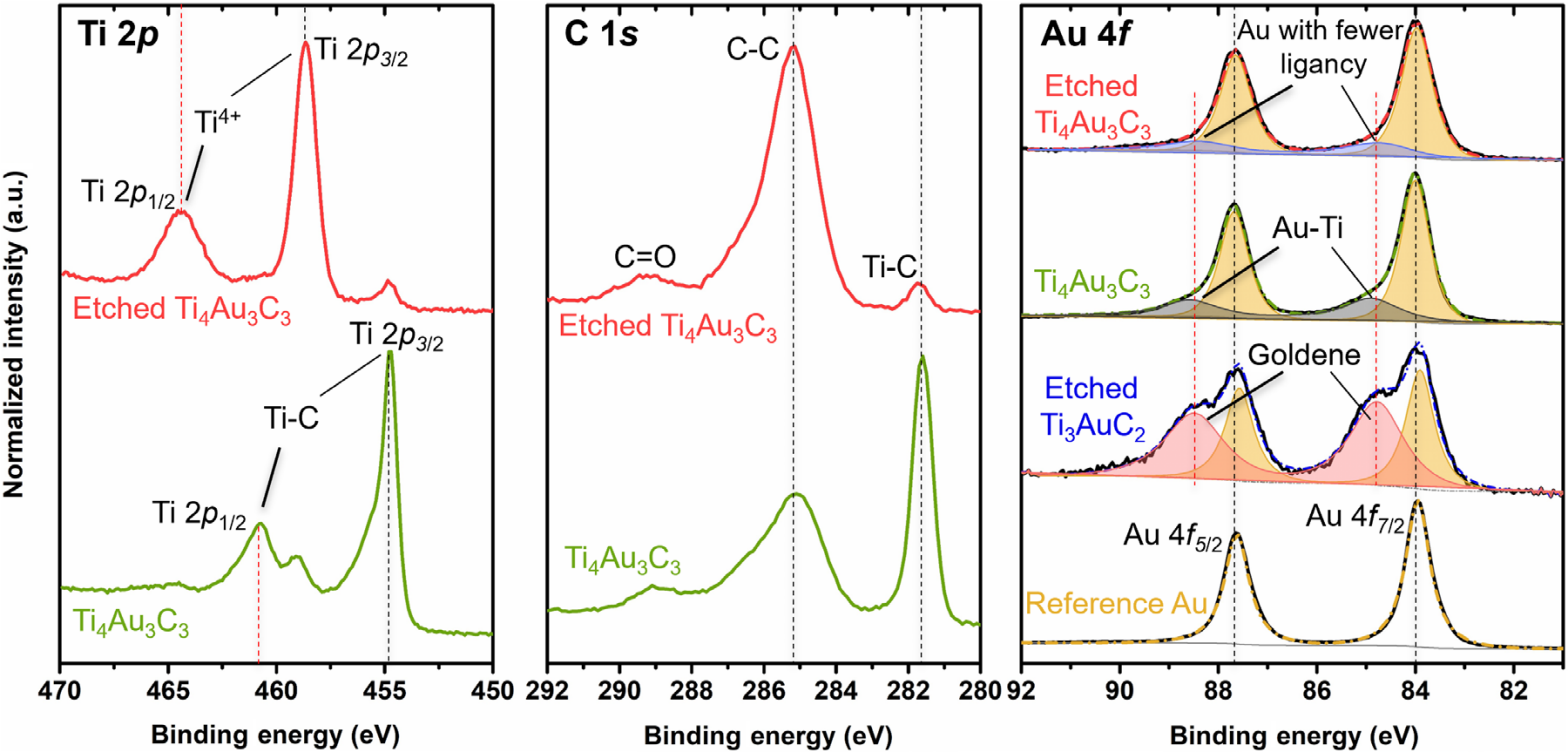
X-ray photoelectron spectra of the trilayer goldene, Ti_4_Au_3_C_3_, monolayer goldene, and reference Au. Ti 2*p* (**left**), C 1*s* (**middle**), and Au 4*f* (**right**) core level spectra measured on the reference sputter-cleaned Au foil (yellow), Ti_4_Au_3_C_3_ (green), etched Ti_4_Au_3_C_3_ (red), and etched Ti_3_AuC_2_ (blue) (*1*). Black solid and colored dash-dot lines represent experimental data and the sum of fitted peaks, respectively. Au-Ti in Ti_4_Au_3_C_3_, Au atoms with fewer ligancy in trilayer goldene, monolayer goldene, and Au-Au interactions are shown in gray, blue, pink, and orange, respectively.

We performed XPS measurements on Ti_4_Au_3_C_3_ before and after etching with 0.5% Murakami’s reagent as well as on a reference sputter-etched Au foil. Figure 4 shows the corresponding Ti 2*p*, C 1*s*, and Au 4*f* core-level XPS spectra. The Au 4*f*_7/2_ peak of the pure reference Au film (Fig. 4, right) is located at 84.0 eV with a 4*f* spin-orbit splitting of 3.7 eV, consistent with reference values (*42*). The Au 4*f*_7/2_ and 4*f*_5/2_ peaks of the unetched Ti_4_Au_3_C_3_ film are asymmetric, revealing the presence of a second low-intensity doublet located at binding energies (*E*_b_) of 84.9 eV and 88.6 eV, respectively, i.e., shifted by 0.9 eV to higher *E*_b_ with respect to the *E*_b_ of the reference Au metal (84.0 and 87.7 eV). The occurrence of the high *E*_b_ doublet is attributed to electronic charge transfer primarily from the Au 5*d* states to the Ti_4_C_3_ sheets in Ti_4_Au_3_C_3_. Similar effects were observed in the Ti_3_AlC_2_ and Ti_2_AlC MAX phases (*1*, *43*, *44*), where charge transfer takes place from Al to the Ti_*n*+1_C*_n_* (*n* = 1 or 2) sheets. The stronger *4f* doublet in the spectrum from the unetched Ti_4_Au_3_C_3_ film appears at nearly the same *E*_b_ as for the reference Au. This can be attributed to the partially remaining capping Au layer on top of Ti_4_Au_3_C_3_ after chemical-mechanical polishing and/or to Au-Au interactions in the trilayer Au. In contrast to Ti_3_AuC_2_, which has in-plane Au-Au interactions within each Au monolayer and Au-Ti interactions on both sides, in the case of Ti_4_Au_3_C_3_ Au-Au interactions are stronger relative to Ti-Au resulting in reduced charge transfer and a higher intensity of the main Au 4*f* doublet with respect to the high *E*_b_ pair.

The etched Ti_3_AuC_2_ film exhibits high *E*_b_ shoulders, shifted by ~0.9 eV with respect to the main Au 4*f* doublets. These shoulders are attributed to final state screening and charge transfer effects (*1*). After etching, the Au 4*f* spectrum of the trilayer goldene, shown at the top of Fig. 4, reveals that the 4*f* doublet peaks are nearly at the same energy positions as those of the reference Au. In addition, a less intense high-energy tail is observed, which can be fitted with a second 4*f*_7/2_-4*f*_5/2_ doublet shifted to higher *E*_b_ values of 84.8 and 88.5 eV, respecitvely. Such *E*_b_ shifts in the high-energy tails of Au 4*f* spectra from monolayer goldene have been attributed to the final state effects (*45*, *46*). It has been demonstrated that both the *E*_b_ and the peak width of the Au 4*f* lines increase as the size of Au nano particles and clusters decreases, due to limited screening of the core hole left after photoionization (*47*). In monolayer goldene produced from Ti_3_AuC_2_ (Fig. 4), the final state effects are enhanced because of the smaller coordination number of Au atoms (six or fewer) compared to bulk *fcc* Au and Ti_3_AuC_2_ (*1*). In contrast, in trilayer goldene, the coordination number of the middle layer is 12, while the outerlayers have a coordination number of 9, likely decreasing further at the sheet edges. Therefore, the final state effects are weaker in trilayer goldene compared to the monolayer, resulting in a less intense tail and a slightly smaller *E*_b_ shift. The dominant Au 4*f* doublet in the spectrum from etched Ti_4_Au_3_C_3_ may originate from residuals of the capping Au layer or from sheets clustering and their curling-up. The tendency of goldene sheets to transform to 3D shapes can be expected from the surface morphology on the Ti_4_Au_3_C_3_ after etching (section S9).

The C 1*s* XPS spectrum of the etched sample (Fig. 4, middle) reveals that the intensity of the carbide peak at ~281.7 eV has markedly decreased as compared to the unetched sample. This indicates that almost all Ti_4_C_3_ has been removed during the etching of Ti_4_Au_3_C_3_. The corresponding Ti 2*p* spectra (Fig. 4, left) further confirm this conclusion: The Ti 2*p*_3/2_-Ti 2*p*_1/2_ peaks due to the carbide (at 454.8 and 460.8 eV, respectively) are very weak in the spectrum from the etched sample. The latter is dominated by a doublet peaks located at 458.6 and 464.5 eV, respectively, i.e., indicative of TiO_2_ formation on the Au layers. Minor impurity peaks from Fe 2*p*, Br 3*d*, and K 2*p* photoemissions (*48*) imply that a small amount of iron and potassium residue from the etchant occurs at the surface after etching (section S10).

## DISCUSSION

We propose that, in addition to monolayer (*1*) and trilayer goldene (present work), isolated bilayer goldene can also be obtained by etching Ti_3_Au_2_C_2_, a compound reported in 2017 (*25*). Combined with the recently reported goldene (*1*, *25*), this work demonstrates that the thickness of isolated Au sheets can be tuned into one-, two-, and three-atomic layers using our devised etching scheme, which includes the use of surfactants. This approach provides a foundation for future fundamental and applied studies on ultrathin Au with varying thicknesses. When using a MAX phase precursor with a larger interlayer distance, such as Ti_4_SiC_3_, there is a greater likelihood that surfactants will penetrate in-between the A layers and stabilize them during exfoliation. Furthermore, by selecting appropriate exchangeable elements, the A layers can be substituted with other noble metals such as silver, platinum, or iridium, leading to the formation of both rising MAX phases and their corresponding metallenes through selective removal of the Ti_*n*+1_C*_n_* slabs.

In conclusion, Ti_4_Au_3_C_3_ MAX phase forms through a solid-state substitution reaction during annealing of Au-covered Ti_4_SiC_3_ thin films. In this process, each Si layer is replaced by three atomic layers of Au, resulting in a 32.5% *c*-axis lattice expansion compared to the original Ti_4_SiC_3_. The insertion of tripple Au layers into Ti_4_SiC_3_ is thermodynamically more stable than the insertion of one or two layers, whereas four and five layers are less favorable. Trilayer goldene has the magic number for reasons of electronegativity and bonding analysis, which identifies Ti_4_Au_3_C_3_ as the optimum structure. Isolated three-atomic-layer Au sheets—trilayer goldene—were obtained by selectively removing the Ti_4_C_3_ sheets from Ti_4_Au_3_C_3_ through wet-chemical etching. Benefiting from the coexistence of *fcc* and *hcp* structures with out-of-plane Au-Au interactions, the trilayer goldene, with a thickness of 6.7 Å, is sufficiently stable to reach a lateral size of up to 100 nm and potentially up to several micrometers, corresponding to the single-crystal domain size in present-day Ti_4_Au_3_C_3_ material. The Au 4*f* binding energy increases by approximately 0.80 eV compared to bulk Au, likely due to the reduced coordination numbers of the surface layers, resulting in a pronounced final state effect. Along with previously reported monolayer goldene, the isolation of gold trilayers in this work suggests that tuning the thickness of quasi-2D Au is feasible, offering research opportunities for exploring physical phenomena and applications based on diverse planar Au nanostructures.

## MATERIALS AND METHODS

Ti_4_SiC_3_ thin films were deposited onto Al_2_O_3_(0001) substrates in a high-vacuum chamber using direct current magnetron sputtering (DCMS) with Ti (99.9%), Si (99.9%), and graphite (99.999%) targets. Before depositions, the substrates underwent a thorough cleaning process, including ultrasonic cleaning in acetone and isopropyl alcohol, followed by rinsing in deionized water. Substrates were then dried under a stream of nitrogen gas. The chamber was maintained at a base pressure of 1 × 10^−10^ torr and filled with Ar of 5 × 10^−5^ torr during all depositions. The substrates were preheated to 900°C at a rate of 25°C/min and kept at constant temperature during the depositions. To assist the Ti-Si-C MAX phase nucleation, a 20-nm-thick TiC seed layer was deposited for 8 min at 900°C with the applied power of 90 and 200 W for Ti and C targets, respectively. The TiC growth was then interrupted by a shutter; meanwhile, the Si magnetron was switched on with an applied power of 30 W. After 1 min, when the targets were stable, the shutter was removed, and Ti-Si-C deposition was initiated. The as-grown Ti_4_SiC_3_ films were around ~100 nm thick with 30-min deposition.

For the Au intercalation, a 200-nm-thick Au layer was sputter-deposited onto the MAX phase film by a separate DCMS system from an Au target (99.99%) under a base pressure of ~5 × 10^−6^ torr. The Au-capped Ti_4_SiC_3_ samples were subsequently annealed in a vacuum annealing furnace at 670°C for 30 hours and 600°C for 8 hours. The quartz tube in the furnace was outgassed a few times before the annealing. The ramping rate was ~5°C/min, and a nitrogen gas flow was introduced during the annealing to avoid oxidation.

Before etching away Ti_4_C_3_ sheets, the residual Au capping layer with a thickness of up to 200 nm was removed by chemical-mechanical polishing to expose the Ti_4_Au_3_C_3_ film. The chemical-mechanical polishing slurry was prepared by mixing fumed silica (2 g, Sigma-Aldrich), I_2_ (1.2 g, Sigma-Aldrich), KI (12 g, Sigma-Aldrich), citric acid (16 g, Sigma-Aldrich), and trisodium citrate (3.7 g, Sigma-Aldrich) in 200 ml of deionized water. Trilayer goldene was produced by etching the Ti_4_Au_3_C_3_ films for 168 hours using 0.5% Murakami’s reagent with 5 mM CTAB (Merck) as a surfactant under complete darkness (*1*). The 0.5% Murakami’s reagent solution was prepared by adding 5 mg of KOH, 5 mg of K_3_[Fe(CN)_6_], and 36 mg CTAB into 10 ml of H_2_O under highly weak ambient light. CTAB used here can hinder the coalescence of the isolated gold sheets into thicker layers and nanoparticles.

HRSTEM measurements were implemented using the Linköping monochromated double-spherical aberration-corrected FEI Titan^3^ 60-300 microscope operating at 300 kV, equipped with an EDX analysis module. The phase composition, crystal structure, and orientation of the samples were analyzed using XRD in a Philips PW 1820 diffractometer with Cu K_α_ radiation. Surface morphology and chemical composition were examined by scanning electron microscopy and EDX analysis with an LEO 1550 Gemini instrument.

XPS analysis was carried out in Axis Ultra DLD instrument from Kratos Analytical (UK) equipped with a monochromatic Al Kα radiation (1486.6 eV) operating at 150 W under the base pressure below 1.5 × 10^−7^ Pa. High-resolution spectra were recorded at a normal emission angle with a pass energy of 20 eV. The spectrometer was calibrated by testing positions of Au 4*f*_7/2_, Ag 3*d*_5/2_, and Cu 2*p*_3/2_ peaks from sputter-etched Au, Ag, and Cu samples in comparison with the recommended ISO (International Organization for Standardization) standards for monochromatic Al Kα sources (*42*). Samples were stored in a glovebox filled with Ar and transferred to the XPS chamber with an Ar-filled sample box before analysis. No Ar etching was carried out before spectra acquisitions. All spectra were charge-corrected to the Fermi edge (section S10).

## Supplementary Materials

### This PDF file includes:

Sections S1 to S11 Figs. S1 to S28 Tables S1 to S6 References
