# Supplementary material for: Synthesis of Ti4Au3C3 and its derivative trilayer goldene through chemical exfoliation
Source: Sci Adv. 2025 Mar 28;11(13):eadt7999. doi: 10.1126/sciadv.adt7999 (PMC11952097; doi:10.1126/sciadv.adt7999)
Supplement: Supplementary file 1 — Sections S1 to S11 Figs. S1 to S28 Tables S1 to S6 References [file sciadv.adt7999_sm.pdf]

Science Advances
AAAS

# Supplementary Materials for

## Synthesis of $Ti_4Au_3C_3$ and its derivative trilayer goldene through chemical exfoliation

Yuchen Shi *et al.*

Corresponding author: Yuchen Shi, tlhsyc2008@gmail.com; Lars Hultman, lars.hultman@liu.se

*Sci. Adv.* **11**, eadt7999 (2025)
DOI: 10.1126/sciadv.adt7999

**This PDF file includes:**

Sections S1 to S11
Figs. S1 to S28
Tables S1 to S6
References

## Section S1: Formation of $Ti_4Au_3C_3$ phase

**Fig. S1** shows an intercalation frontline in the annealed Au-covered $Ti_4Au_3C_3$ film. It can be seen in panels A and B that three layers of Au follow closely to substitute Si and expand the lattice along the *c*-axis (red square). The bending of $Ti_4C_3$ sheets, while remaining intact, is apparent when Au layers enter, as indicated by blue lines. It is known that defects such as grain boundaries and dislocations provide paths for the in-diffusion of Au (*25*, *49*), as shown in panel C. Thus, exchange intercalation of Si and Au progresses both vertically and laterally in MAX phases.

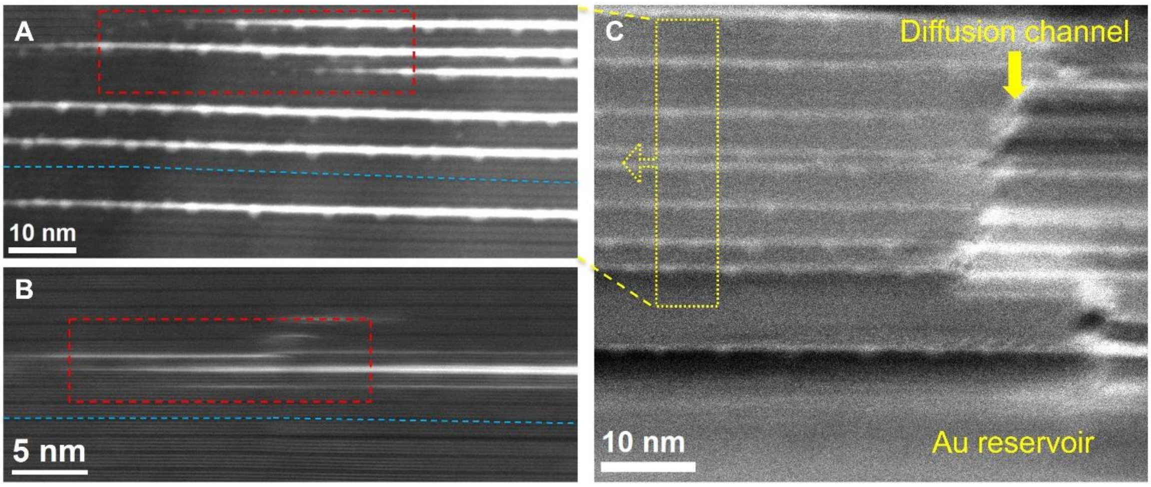


**Fig. S1. Intercalation frontline of Au in $Ti_4SiC_3$.** (**A** and **B**) HRSTEM images of an intercalation frontline of three-atomic-layers Au, where two additional Au layers are inserted closely behind the first layer, as squared by red dashed lines. The blue dashed lines indicate that $Ti_4C_3$ sheets suffer from vast deformation due to the insertion of three atomic layers of Au, resulting in a large lattice expansion. (**C**) A dislocation region that allows Au to in-diffuse. Panel (A) is taken from the further left area, indicated by the dotted yellow arrow.

Regarding the substitution mechanism, Au capping layer induces instability in the bonding character of the Si adjacent to the Au in Au-Si diffusion system by screening of the Coulomb interaction by its free electrons (*25*, *50*). The annealing temperatures of 600-670 °C in this work are higher than the eutectic point at 363 °C of the Au–Si binary system. Liquid phase is dominant throughout the whole phase diagram at high temperatures, which can promote the diffusion of two elements. The loosely bonded Si atoms that are provided with a reduced chemical-potential path to diffuse out into the Au capping layer, leaving behind

vacancies that are subsequently backfilled with Au. Grain boundaries, dislocations, and stacking faults are the most likely diffusion channels for promoting substitution reactions.

Thus, the substitution reaction can be modeled as a two-step process, step 1 being the out-diffusion of the original A element (Si) into the capping layer A’ (Au), thereby leaving vacancies in the A layer behind, and step 2 the diffusion of A′ atoms into the A layer to backfill the vacancies subsequently. The number of A’ layers inserted depends on the stability of different structures after thermal intercalation.

## Section S2: Formation of $Ti_4AuC_3$ phase

To explore the possibility of producing the $Ti_4AuC_3$ phase, we annealed Au-covered $Ti_4SiC_3$ samples at a lower temperature of 600 °C with a duration of 8 h under a nitrogen gas flow. The STEM image in **Fig. S2A** shows that the Si layers on the A sites were substituted with monolayer Au, which are periodically mirrored with respect to the $Ti_4C_3$ sheets. The *c*-parameter increased from 22.8 Å for $Ti_4SiC_3$ to 23.6 Å for $Ti_4AuC_3$ with a 3.5 % lattice swelling. This value is close to a calculated c-parameter for $Ti_4AuC_3$ of 23.621 (see **Table S1**) and 23.578 Å (*25*). The EDX spectrum in **Fig. S2B** shows the signal of Ti, Au, and Si with a relative atomic ratio of Ti:(Au+Si) is ∼4:1, consistent with a stoichiometric 413 MAX phase. However, this phase exists only a few nanometers along the *c*-axis and was found very close to the TiC seed layer. It was sandwiched by TiC and other stable phases ($Ti_3AuC_2$ in most cases, $Ti_7Au_2C_5$ and excess Au), as seen in **Fig. S3**. As aforementioned, sufficient space is needed for the lattice expansion (+42.5 %) to form the $Ti_4Au_3C_3$ phase. These stable phases might limit the lifting space of $Ti_4C_3$ sheets and impede the insertion of additional Au layers; as a result, the $Ti_4AuC_3$ phase is confined and stabilized. Among them, the $Ti_3AuC_2$ phase originated from Au intercalation in $Ti_3SiC_2$ phase, which is a transition layer when Ti-Si-C nucleated on the TiC seed layer at the initial stage of deposition. The $Ti_7Si_2C_5$ phase also formed at the initial stage when the ratio of target elements was undulated. Wherein, each Si layer was substituted with single-layer Au, forming the $Ti_7Au_2C_5$ phase (**Fig. S3B**). **Fig. S2C** shows the XRD patterns of this sample before and after annealing. The typical (000*l*) peaks of the $Ti_4Au_3C_3$ phase are observed at the same diffraction angles as those in **Fig. 1E** (main text), whereas the intensities of these peaks are much lower due to less intercalation of Au. The (000*l*) peaks of the $Ti_3AuC_2$ phase are found at 9.8°, 19.2° and 29.3°, while the $Ti_4AuC_3$ phase is hardly detectable. We gradually decreased the duration from 16 h to 1 h of annealing at 670 °C for Au-covered $Ti_4SiC_3$ films and investigated the impact of annealing temperatures in the range of 580-670 °C with a constant duration of 8 h. The majority within these films is always $Ti_4Au_3C_3$ under such annealing conditions (**Figs. S4** and **S5**). The substitution reaction cannot be driven for annealing temperatures below 580 °C.

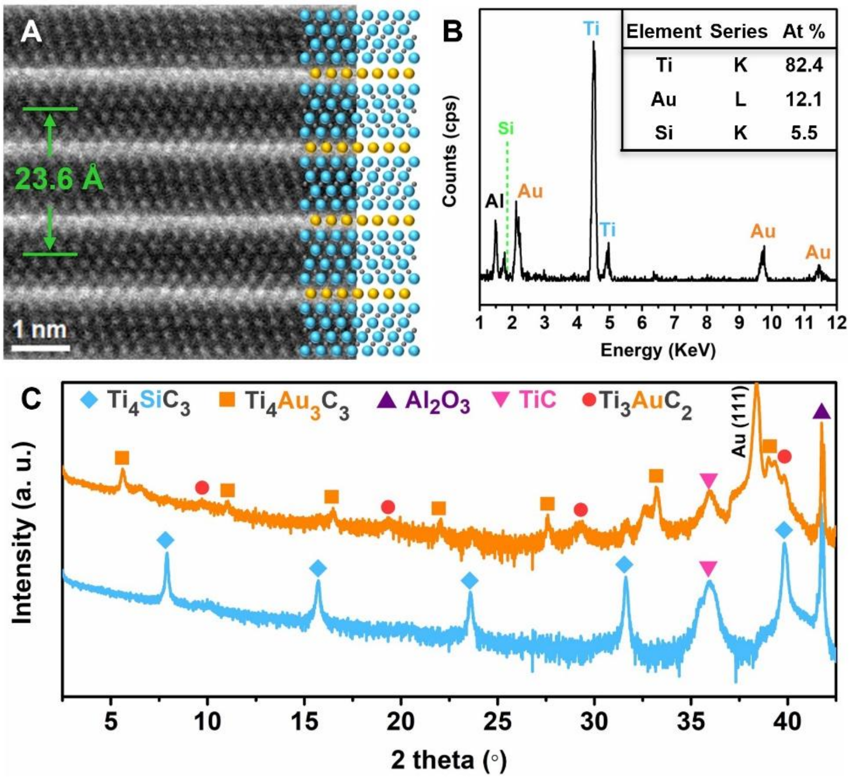


**Fig. S2. Synthesis of $Ti_4AuC_3$ phase.** (**A**) HRSTEM image of $Ti_4AuC_3$ taken along the $[11\bar{2}0]$ direction. Blue, yellow, and dark gray balls represent Ti, Au, and C atoms, respectively. (**B**) EDX spectrum of $Ti_4AuC_3$ phase showing atomic ratio of Ti, Au, and C elements. (**C**) XRD patterns of $Ti_4SiC_3$ and Au-covered $Ti_4SiC_3$ after 8 h of annealing at 600 °C.

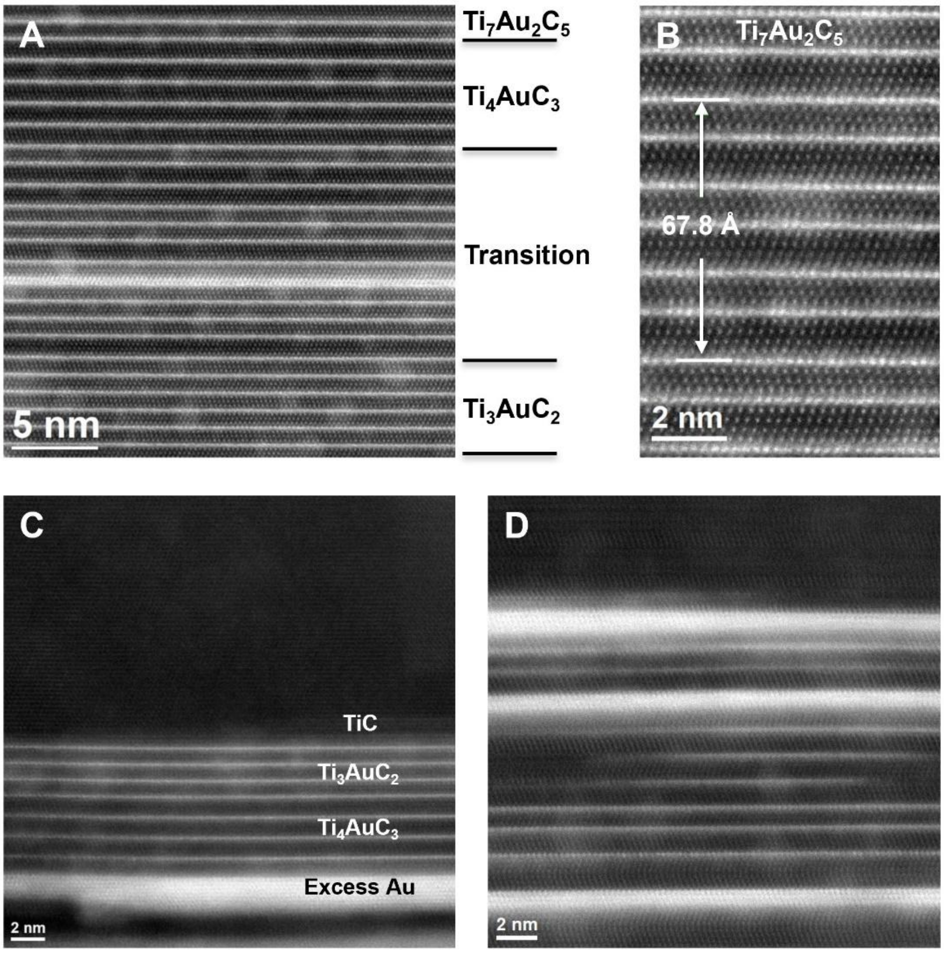


**Fig. S3. Formation of $Ti_4AuC_3$ in-between other stable phases.** (**A**) HRSTEM image showing a $Ti_4AuC_3$ phase that is sandwiched by $Ti_7Au_2C_5$, $Ti_3AuC_2$, and a transition region consisting of a mixture of $Ti_4AuC_3$ and $Ti_3AuC_2$ subunits. (**B**) Zoomed-in $Ti_7Au_2C_5$ phase formed from $Ti_7Si_2C_5$ via substitution reaction after 8 h of annealing at 600 °C. (**C** and **D**) Examples for $Ti_4AuC_3$ being sandwiched by $Ti_3AuC_2$, TiC, and intercalated excess Au.

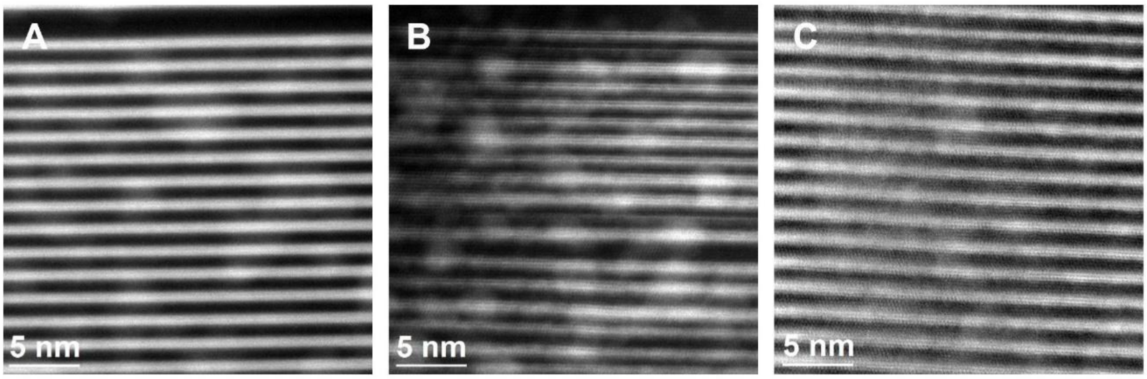


**Fig. S4. Formation of $Ti_4Au_3C_3$ with various annealing durations.** HRSTEM images of Au-covered $Ti_4SiC_3$ annealed at 670 °C for 12 h (**A**), 8 h (**B**), and 1 h (**C**), respectively. All images were recorded along the $[11\bar{2}0]$ direction.

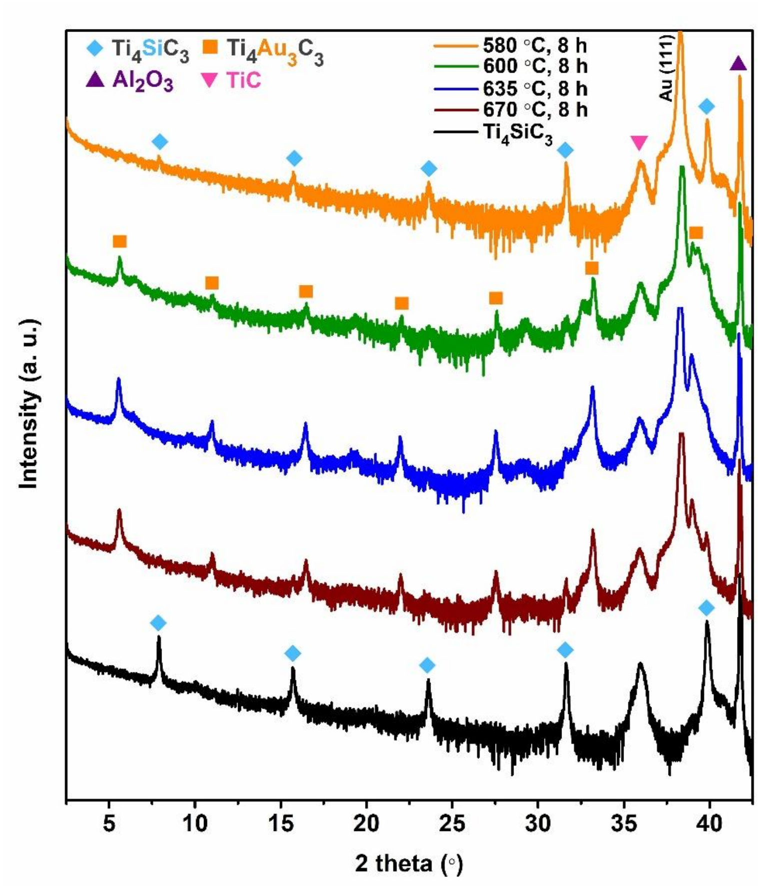


**Fig. S5. XRD patterns of Au-capped $Ti_4SiC_3$ after 8 h of annealing at different temperatures in the range of 580-670 °C.**

## Section S3: DFT computations for $Ti_4Au_{x+1}C_3$ phases

The structural relaxation was performed in terms of density functional theory (DFT) calculations using the Vienna *Ab initio* Simulation Package (VASP) (*51-53*) with the Perdew–Burke–Ernzerhof (*54*) generalized gradient approximation for the exchange-correlation functional. We use Materials Project (*55*) recommended projector augmented-wave atom potentials for all elements and a plane-wave cutoff energy of 520 eV for the plane-wave basis set. Reciprocal space sampling was performed using a 0.05 $Å^{-1}$ *k*-point density. The total energy is minimized through the relaxation of unit-cell shape and volume, and internal atomic positions until satisfying an energy convergence of $10^{-7}$ eV/atom and a force convergence of $10^{-2}$ eV/Å. Owing to the use of consistent calculation settings, the calculated energies can be directly compared with data in the Materials Project database.

The energy cost or gain by inserting extra Au layers into $Ti_4AuC_3$ was calculated using

$$\Delta E_{\mathrm{Ti_4AuC_3}} = E_{\mathrm{Ti_4Au_{1+\mathit{x}}C_3}} - E_{\mathrm{Ti_4AuC_3}} - xE_{Au}, \tag{S1}$$

where the terms $E_{\mathrm{Ti_4Au_{1+\mathit{x}}C_3}}$, $E_{\mathrm{Ti_4AuC_3}}$, $E_{Au}$ represent the calculated total energy of $Ti_4Au_{1+x}C_3$, $Ti_4AuC_3$, and Au in their relaxed bulk crystal structure, and *x* is the number of added Au layers.

Stability for $Ti_4Au_{1+x}C_3$ has been evaluated in terms of formation enthalpy, $\Delta H_{\mathrm{cp}}$, by comparing its calculated energy with respect to the energy of known and hypothetical competing phases within the Ti-Au-C system. Competing phases considered herein include compounds available in the Materials Project database (*55*), $Ti_{n+1}Au_{1+x}C_n$ compositions with *n* and *x* = 1, 2, and 3, considered in Ref. (*25*, *27*) and in this work. To find the combination of competing phases having the lowest energy we apply a linear optimization procedure based on the simplex method. Further details are given in Ref. (*56*, *57*).

Chemical bonding was investigated in terms of crystal orbital Hamiltonian populations (COHP) which were derived using the LOBSTER program (*58-60*). With this approach, the calculated band-structure energy is reconstructed into orbital interactions. Positive COHP values indicate antibonding interactions while negative COHP values indicate bonding interactions. To facilitate interpretation and to preserve the analogy for crystal orbital overlap population (COOP) analysis, the results are here presented as −COHP. Visualization of atomic structures was done with the VESTA code (*61*).

**Figs. S6-S10** show multiple structures when inserting Au layers into $Ti_4SiC_3$ including different stackings of the Au layers as well as different stackings of the $Ti_4C_3$ subunits with respect to the Au layers. Calculated lattice parameters together with space group symmetry and stability for $Ti_4Au_{1+x}C_3$ phases are listed in **Tables S1** and **S2**. **Fig. S11** illustrates the bonding analysis for different $Ti_4Au_{1+x}C_3$ structures.

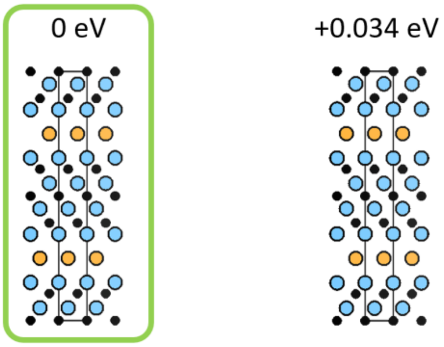


**Fig. S6. Structures considered for $Ti_4AuC_3$ with Ti in blue, Au in gold, and C in black.** Green rectangle mark structure used for analysis in **Fig. 2**. Energies given are calculated by using Eq. S1.

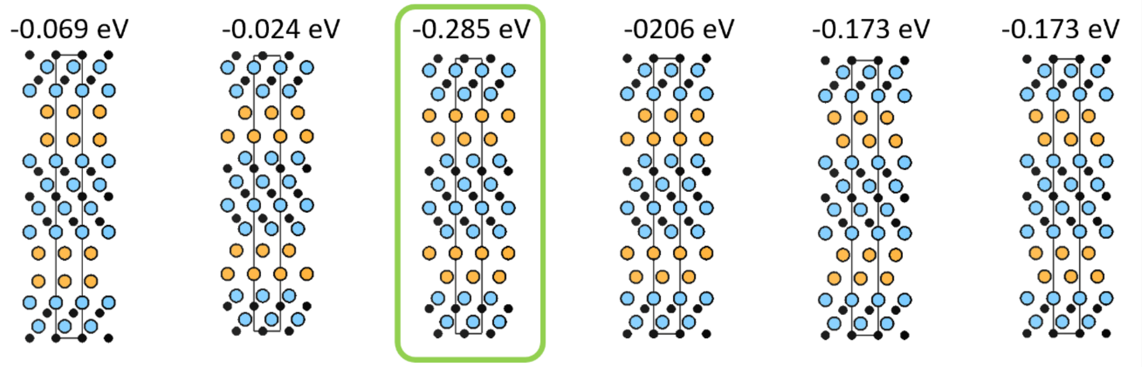


**Fig. S7. Structures considered for $Ti_4Au_2C_3$ with Ti in blue, Au in gold, and C in black.** Green rectangle mark structure used for analysis in **Fig. 2**. Energies given are calculated by using Eq. S1.

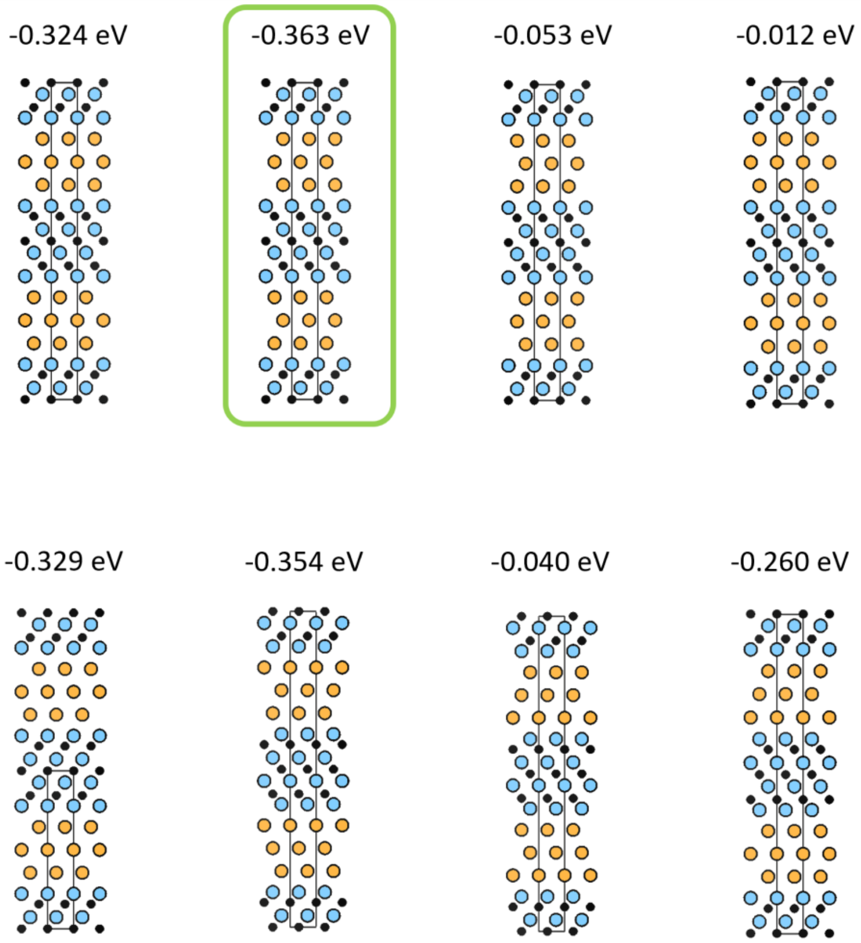


**Fig. S8. Structures considered for $Ti_4Au_3C_3$ with Ti in blue, Au in gold, and C in black.** Green rectangle mark structure used for analysis in **Fig. 2**. Energies given are calculated by using Eq. S1.

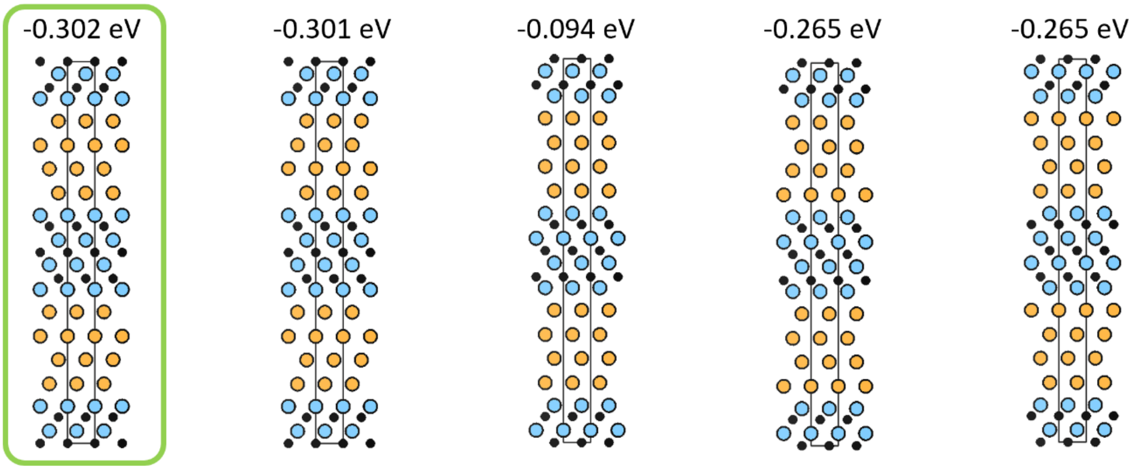


**Fig. S9. Structures considered for $Ti_4Au_4C_3$ with Ti in blue, Au in gold, and C in black.** Green rectangle mark structure used for analysis in **Fig. 2**. Energies given are calculated by using Eq. S1.

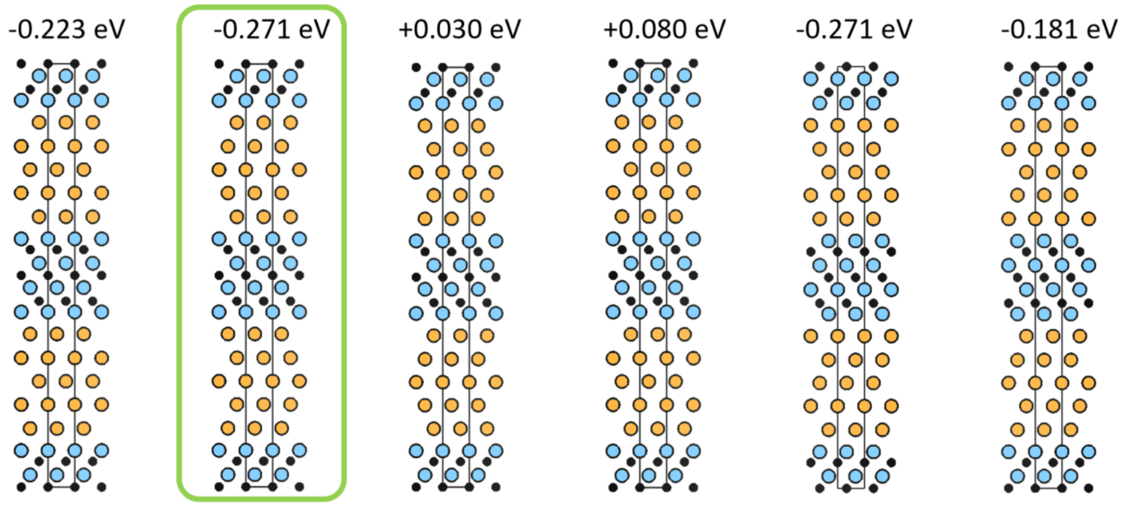


**Fig. S10. Structures considered for $Ti_4Au_5C_3$ with Ti in blue, Au in gold, and C in black.** Green rectangle mark structure used for analysis in **Fig. 2**. Energies given are calculated by using Eq. S1.

**Table S1. Calculated lattice parameters, space group symmetry and Wyckoff sites for structures illustrated in Fig. 2A.**

| Phase | *a* (Å) | *c* (Å) | Space group symmetry | Wyckoff sites |
|---|---|---|---|---|
| $Ti_4AuC_3$ | 3.087 | 23.621 | $P6_3/mmc$ (no. 194) | Ti @ 4e, 4f<br>Au @ 2d<br>C @ 2a, 4f |
| $Ti_4Au_2C_3$ | 3.096 | 27.964 | $P6_3mc$ (no. 186) | Ti @ 2a, 2b, 2b, 2b<br>Au @ 2a, 2b<br>C @ 2a, 2b, 2b |
| $Ti_4Au_3C_3$ | 3.090 | 32.553 | $P6_3/mmc$ (no. 194) | Ti @ 4e, 4f<br>Au @ 2c, 4f<br>C @ 2a, 4f |
| $Ti_4Au_4C_3$ | 3.081 | 37.225 | $P6_3mc$ (no. 186) | Ti @ 2a, 2a, 2b, 2b<br>Au @ 2a, 2a, 2b, 2b<br>C @ 2a, 2b, 2b |
| $Ti_4Au_5C_3$ | 3.076 | 41.829 | $P6_3/mmc$ (no. 194) | Ti @ 4e, 4f<br>Au @ 2b, 4f, 4f<br>C @ 2a, 4f |

**Table S2. Phase stability calculated for $Ti_4Au_{1+x}C_3$, in terms of formation enthalpy $\Delta H_{cp}$, with three different sets of competing phases.**

| Phase | Unaries and binaries | | Unaries, binaries and ternaries ($Ti_4Au_{1+x}C_3$ excluded) | | Unaries, binaries and ternaries ($Ti_4Au_{1+x}C_3$ included) | |
|---|---|---|---|---|---|---|
| | $\Delta H_{cp}$ (meV/atom) | Most competing phases | $\Delta H_{cp}$ (meV/atom) | Most competing phases | $\Delta H_{cp}$ (meV/atom) | Most competing phases |
| $Ti_4AuC_3$ | -41 | TiC, TiAu | -2 | TiC, $Ti_3AuC_2$ | -2 | TiC, $Ti_3AuC_2$ |
| $Ti_4Au_2C_3$ | -22 | TiC, TiAu2 | 0 | TiC, $Ti_3Au_2C_2$ | 0 | TiC, $Ti_3Au_2C_2$ |
| $Ti_4Au_3C_3$ | -20 | TiC, $TiAu_2$, $TiAu_4$ | -2 | TiC, $Ti_3Au_3C_2$ | -2 | TiC, $Ti_3Au_3C_2$ |
| $Ti_4Au_4C_3$ | -7 | TiC, TiAu4 | 4 | TiC, Au, $Ti_3$Au3$C_2$ | 6 | Au, $Ti_4Au_3C_3$ |
| $Ti_4Au_5C_3$ | -3 | TiC, TiAu4, Au | 7 | Au, TiC, $Ti_3Au_3C_2$ | 8 | Au, $Ti_4Au_3C_3$ |

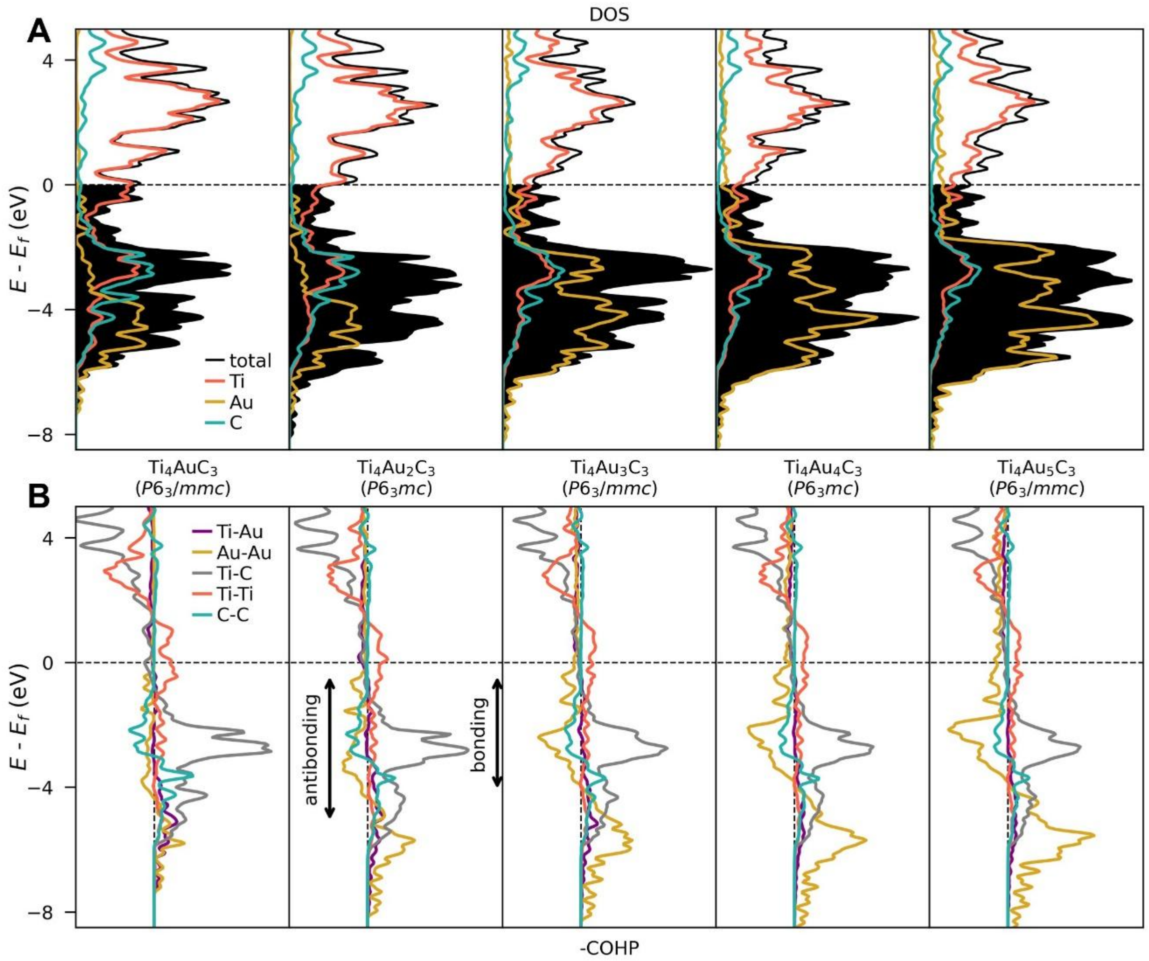


**Fig. S11. Bonding analysis for different $Ti_4Au_{1+x}C_3$ structures.** Calculated (**A**) electronic density of states (DOS) and (**B**) COHP curves for $Ti_4Au_{1+x}C_3$ structures illustrated in **Fig. 2A** (main text).

## Section S4: Tri-layer stackings of Au in $Ti_4Au_3C_3$ – Why is three layers of goldene more stable than one, two, four or five?

ABA and ABC stand for the stacking sequences of three atomic layers in hexagonal close-packed (*hcp*) structure and close-packed (*fcc*) structure, respectively.

In a *hcp* structure, the third layer has the same arrangement of spheres as the first layer and covers all the tetrahedral holes. Since the structure repeats itself after every two layers, the stacking for *hcp* may be described as 'A-B-A'.

In a *fcc* structure, like *hcp*, the second layer of spheres is placed onto half of the depression of the first layer. The third layer is completely different than that first two layers and is stacked in the depressions of the second layer, thus covering all the octahedral holes. The spheres in the third layer are not in line with those in layer A, and the structure does not repeat until a fourth layer is added. The fourth layer is the same as the first layer, so the arrangement of layers is 'A-B-C'.

**Fig. S12** shows a large-scale STEM image of $Ti_4Au_3C_3$ taken containing some regions of *hcp* and *fcc* trilayers Au. The inset indicates a transition region between *hcp* and *fcc* Au along a trilayer. The Au atoms in the third layer move from a staggered position to a mirrored position with respect to those in the first layer. Also, slight changes in atomic density in the horizontal direction can be observed at the transition.

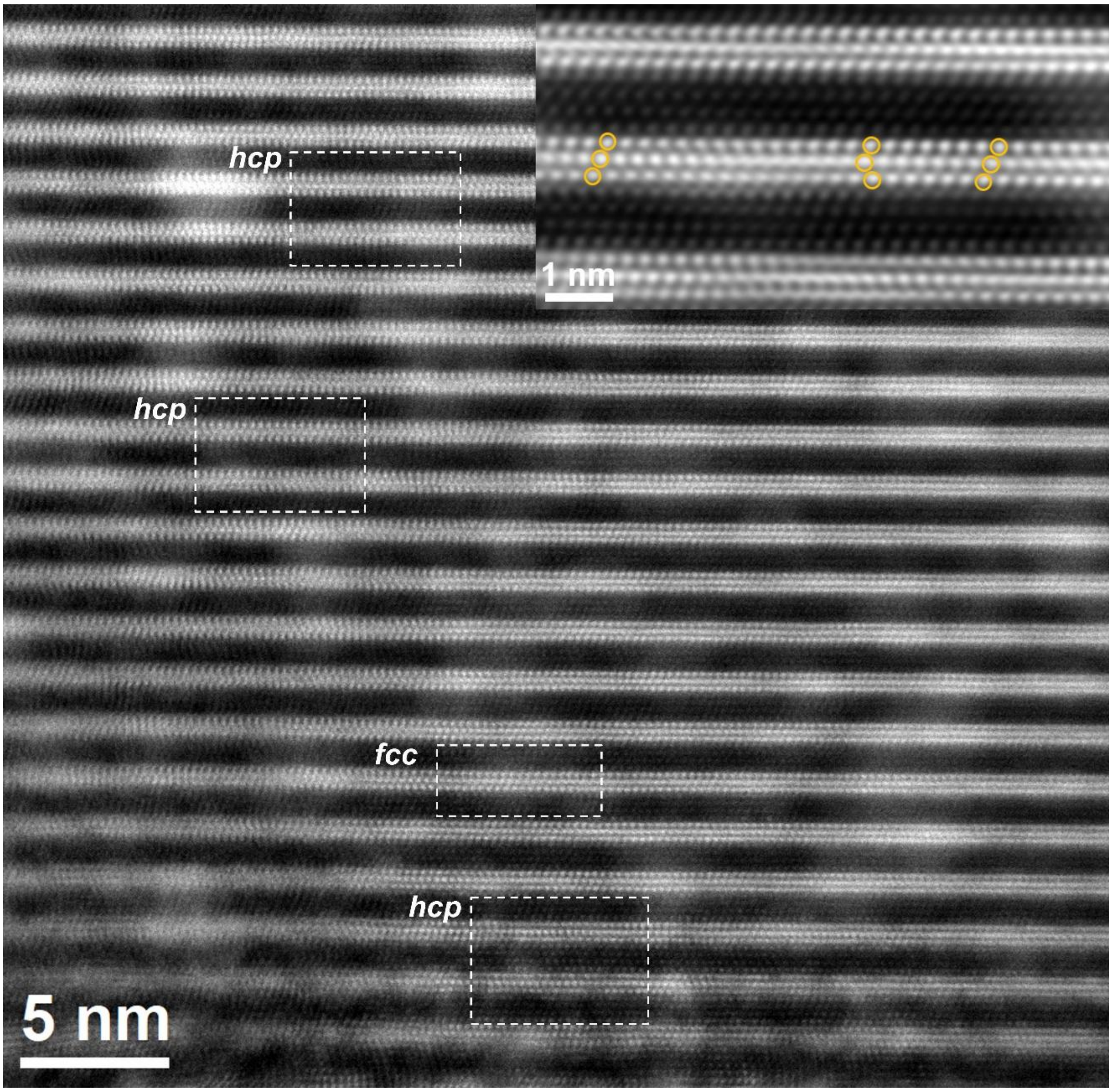


**Fig. S12. 2H-*hcp* and *fcc* structures of trilayers Au in $Ti_4Au_3C_3$.** Large-scale HRSTEM image of $Ti_4Au_3C_3$ taken along the $[11\bar{2}0]$ direction showing both 2H *hcp* Au and *fcc* trilayer Au in-between $Ti_4C_3$ sheets. The inset shows a transition between *hcp* and *fcc* Au along a trilayer, as indicated by yellow circles.

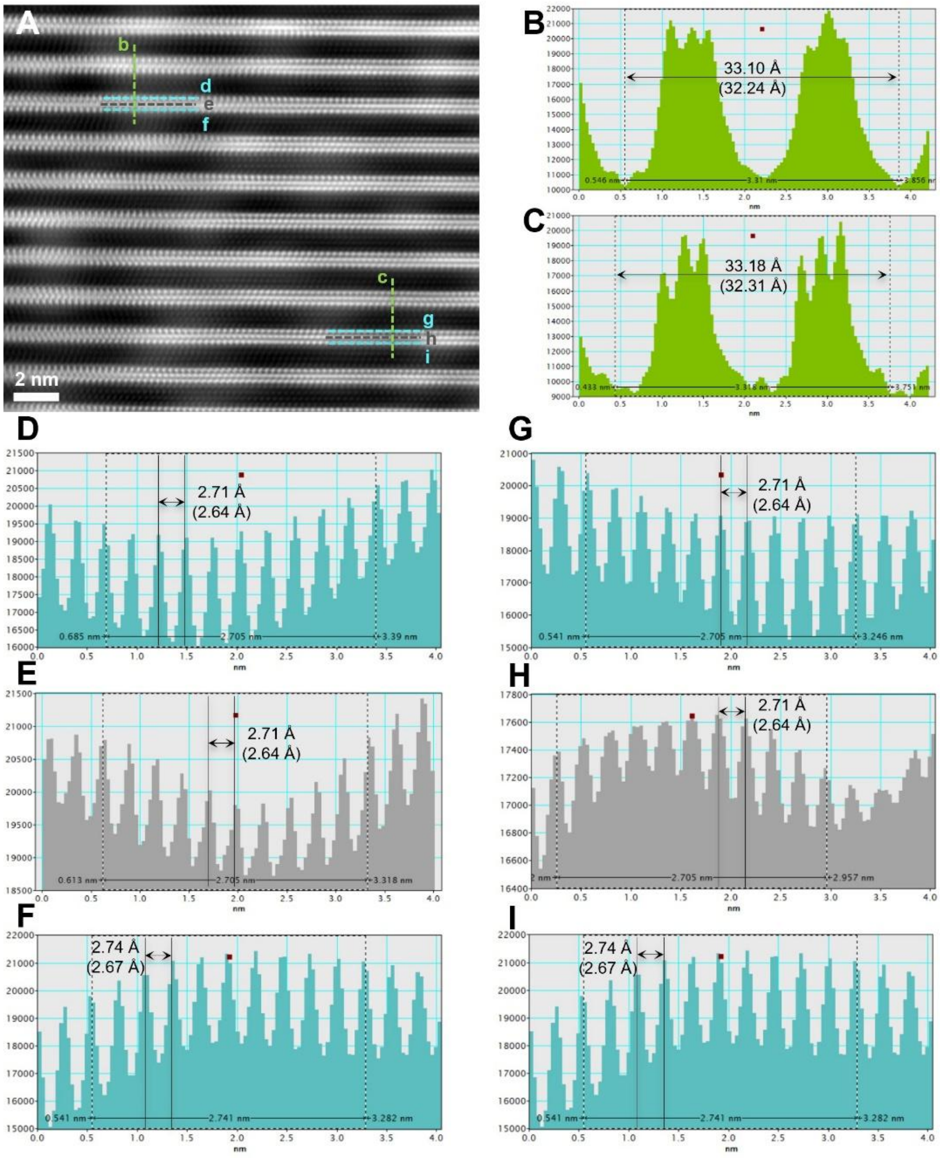


**Fig. S13. In-plane Au-Au spacings within trilayers *hcp* Au and the *c*-parameter of $Ti_4Au_3C_3$ with *hcp* Au.** (**A**) HRSTEM image of $Ti_4Au_3C_3$ along with [11$\bar{2}$0] direction showing two *hcp*-Au regions. (**B** and **C**) The measured *c* lattice parameter of $Ti_4Au_3C_3$ is 33.15 Å on average. The distances were calibrated using the reference *c*-parameter value of 32.30 Å. (**D** to **I**) Line profiles extracted from the dash lines highlighted in (A). Values in parentheses are calibrated distances.

**Table S3. Average distances obtained from line profiles in Fig. S13 and calculated in-plane Au-Au distances.**

| Au layers in $Ti_4Au_3C_3$ | *Calibrated average distances from line profiles (Å) | Calculated in-plane Au-Au distances (Å) |
|---|---|---|
| Next to Ti | 2.65 | 3.06 |
| Middle | 2.64 | 3.05 |

*The distance of two crests in line profiles is the spacing of (1$\bar{1}$0) planes in 2H *hcp* Au, which is equal to $\sqrt{3}/2$ times the in-plane Au-Au distance.

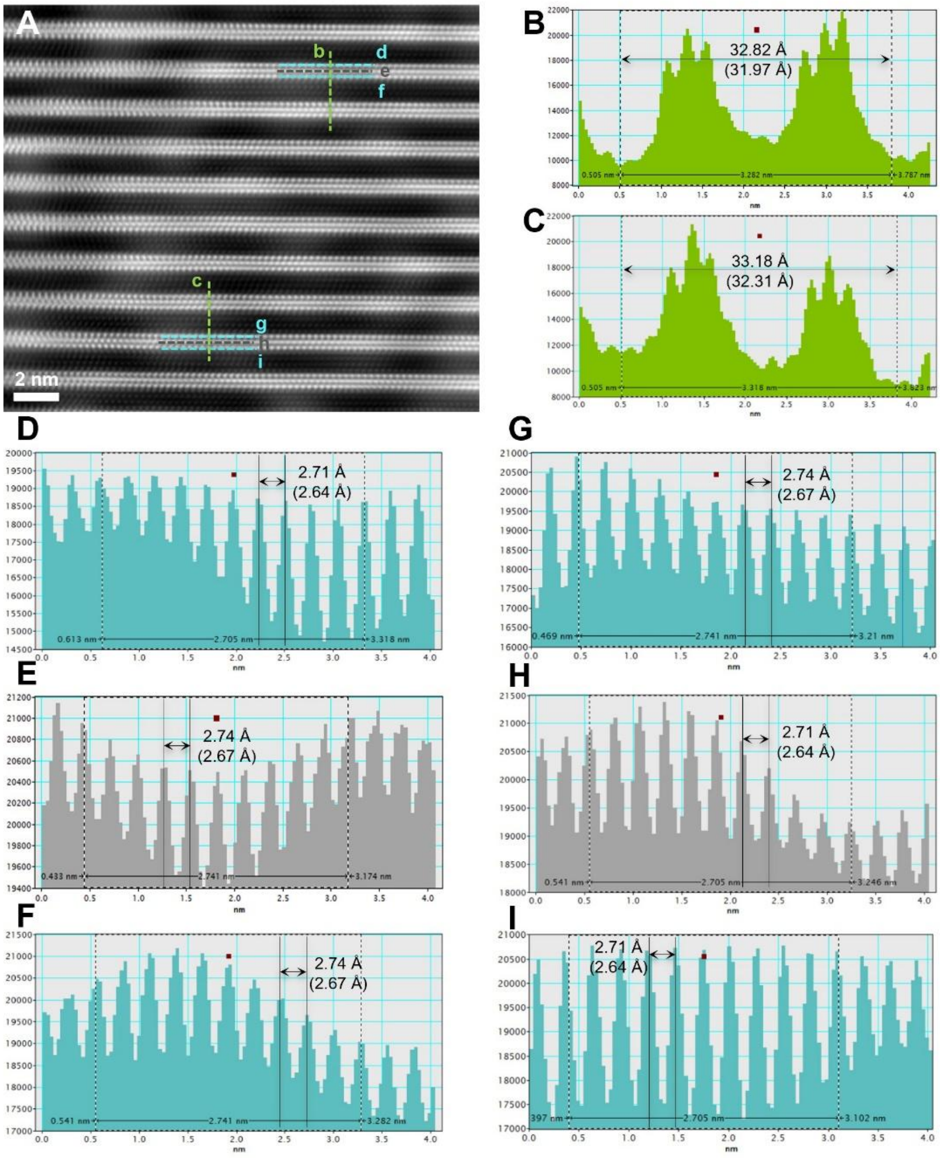


**Fig. S14. In-plane Au-Au spacings within trilayers *fcc* Au and the *c*-parameter of $Ti_4Au_3C_3$ with *fcc* Au.** (**A**) HRSTEM image of $Ti_4Au_3C_3$ along with [11$\bar{2}$0] direction showing two *fcc*-Au regions. (**B** and **C**) The calibrated *c* lattice parameter of $Ti_4Au_3C_3$ is 32.14 Å on average. The distances were calibrated using the reference *c*-parameter value of 32.30 Å. (**D** to **I**) Line profiles extracted from the dash lines highlighted in (A). Values in parentheses are calibrated distances.

**Table S4. Average distances obtained from line profiles in Fig. S14 and calculated in-plane Au-Au distances.**

| Au layers in $Ti_4Au_3C_3$ | *Calibrated average distances from line profiles (Å) | Calculated in-plane Au-Au distances (Å) |
|---|---|---|
| Next to Ti | 2.65 | 3.06 |
| Middle | 2.65 | 3.06 |

*The distance of two crests in line profiles is the spacing of (1$\bar{1}$0) planes in *fcc* Au, which is equal to √3/2 times the in-plane Au-Au distance.

We now examine the relative stability of the tri-layer goldene from the difference in electronegativity between Au (2.54), Ti (1.54), and C (2.55) compared to Si (1.90) or Al (1.61). As Au is more electronegative than Ti, there will be electronic charge transfer from Ti to Au. The stability of goldene stacks would then be a delicate balance of charge-transfer for the metal bonding between the Au atoms and a trilayer of Au happens to cause the “right” amount of charge transfer from Ti to Au for its highest stability, compared to less or more gold layers. A monolayer of Au implies too much charge-transfer from Ti to Au which is less energetically favorable compared to trilayer Au, where the middle Au layer does not attract as much charge as the surface interface Au layers. On the other hand, four and five goldene layers imply less charge transfer to Au which is also less favorable for the stability compared to trilayer goldene. The metal bonding weakens if there is too much charge-transfer from a metal. The Au surface should ideally be equally attractive on both sides as in the case of a trilayer *hcp* or *fcc* goldene containing a unit cell of three layers while a bilayer or other even number of layers of goldene layers shows different surfaces on each side towards the $Ti_4C_3$ slabs that is probably less favorable for this stacked system.

DFT calculation combined with Bader analysis (*62*) was made to study the charge transfer dependence as function of number of goldene layers for the low-energy structures visualized in **Fig. 2A** (main text). **Fig. S15** shows the charge for each layer.

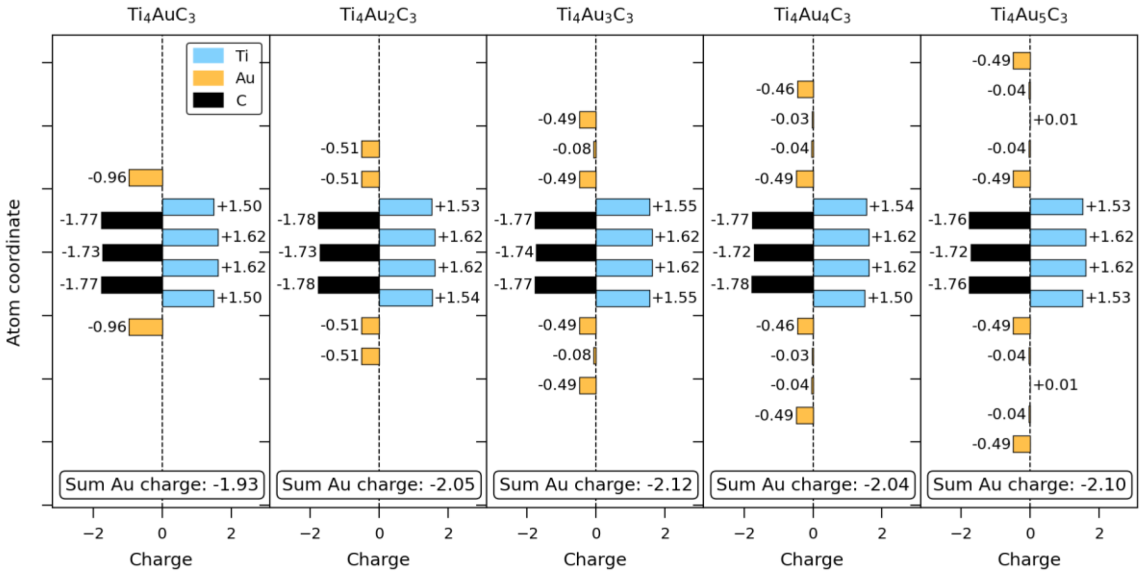


**Fig. S15. DFT test calculations of the charge transfer dependence of atomic layers in $Ti_4Au_{1+x}C_3$ as a function of the number of goldene layers.** The low-energy structures are those visualized in **Fig. 2A** (main text).

Overall, Ti is positive and C negative, which is in line with their electronegativity. Goldene layers next to Ti are always negatively charged while goldene further away from Ti are close to neutral. For Au, we find drastic changes when going from 1 to 2 to 3 (-0.96 to -0.51 to -0.49/-0.08 per goldene layer). For five goldene layers, the central layer is close to neutral. Summing, the charge for the goldene gives the most negative charge for tri-layer goldene. Note that the results for four layers of goldene look a bit strange. This can be related to its structure, which is less symmetric compared to the other ones. This is seen upon a closer look at $Ti_4Au_4C_3$ in **Fig. 2A**. Ti is found to be the most positive for 3 layers of Au. Ti is least impacted when with respect to monolayer goldene. These arguments may explain why three is the optimum number for stacks of goldene, as experimentally observed in TEM.

## Section S5: Mechanistic consideration on $Ti_4C_3$ sheets gliding in $Ti_4Au_3C_3$

The progressive displacement of Ti-C sheets was first noted in $Ti_3Au_2C_2$ when inserting first one and then two Au layers into $Ti_3SiC_2$ (*25*), and an example is given in **Fig. S16**. Such deformation in MAX phases was, however, not explained.

The universal mechanism of plastic deformation and diffusional creep in crystalline materials at elevated temperatures is grain boundary (GB) sliding, sometimes coupled with GB migration (*63*, *64*). MAX phases have two additional deformation mechanisms: first there are basal plane dislocations, which are mobile and able to multiply at room temperature. Confining the dislocations to the basal planes provokes kink band formation (*65*). An incipient kink band (IKB) (*66*) is one in which the walls of opposite sign dislocations are undissociated i.e. still attracted to each other, which ensures that when the load is removed the IKB's would be annihilated. Second, there are ripplocations; in contrast to dislocations, these have no Burgers vector and no polarity but are based on the buckling of surfaces in Van der Waals layers. Thus, Au-intercalated MAX phases are expected to be compliant towards mechanical strain from the c-axis expansion of varying degree as the number of Au layers builds up. Note that the a-axis remains more or less constant, so the registry between A (or A') and MX layers is intact.

In $Ti_4Au_3C_3$, we observed a dislocation structure that often exists in $Ti_4Au_3C_3$ phases (**Fig. S17A**). It should be an IKB since we have not observed separate dislocation walls in extensive samples. The stress that caused this IKB may come from the insertion of additional gold layers from one side (e. g., in the lower right corner of **Fig. S17A**). Thus, the formation and annihilation of IKBs would be reversible when the number of inserted Au layers is consistent. We also observed a ripplocations region (**Fig. S17B**) where the crystal responds to the stress along the basal planes by forming surface buckling or ripplocations. However, this case is not common in our observations. We speculate that the shear stress along the basal planes might be mainly introduced by IKBs (or both IKBs and ripplocations) generated from inserting two additional Au layers together with the first layer, and by an inhomogeneous diffusion channel for Au entering.

A GB slides when the two adjacent grains undergo relative displacement parallel to the boundary plane under shear stress. It was reported experimentally and by molecular dynamics simulations that nano-twinned lamellae in Au nanocrystals undergo detwinning in the opposite direction under shear loading and subsequent extensive sliding along the twin boundary (TB) plane; the nanoscale twins exhibited substantial shear deformability along the TB with increased shear strain (*67*). In $Ti_4Au_3C_3$, the interface between $Ti_4C_3$ sheets and Au sheets can

be regarded as a TB plane due to the perfect symmetry of $Ti_4C_3$ sheets concerning Au. During temperature varies after Au intercalation, two adjacent $Ti_4C_3$ sheets glide along the TB because of weak Ti-Au interactions until the internal stress balances the adventive shear stress. As a result, the system energy is minimized, and a stable $Ti_4Au_3C_3$ phase can form with a zigzag stacking of $Ti_4C_3$ coupled with ABC-stacked Au. As mentioned in the main text, the horizontal gliding of two adjacent $Ti_4C_3$ sheets is necessary since they are mirrored with Si in $Ti_4SiC_3$ but become non-mirrored after the formation of ABC-stacked tri-layers Au. The gliding of $Ti_4C_3$ sheets may be towards the same direction if the stress originates from only one side, i.e., the same direction as the Au insertion.

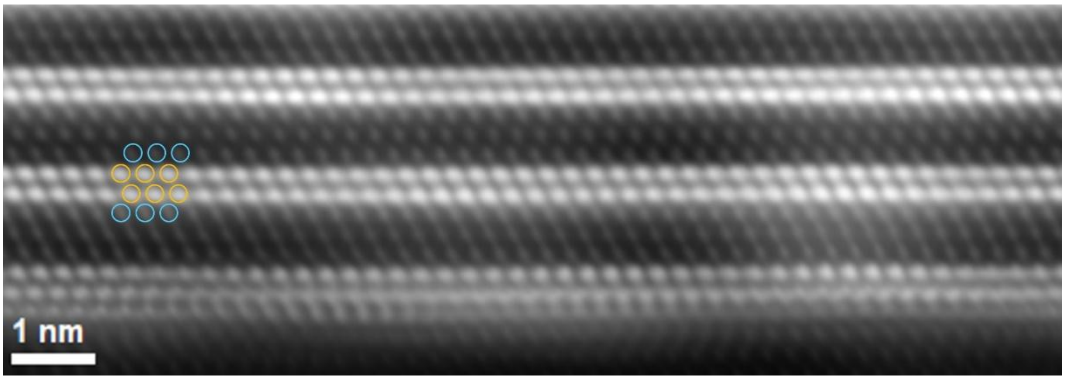


**Fig. S16. Structure of $Ti_3Au_2C_2$.** Cross-sectional HRSTEM image of $Ti_3Au_2C_2$ along with [$11\bar{2}0$] direction. Ti and Au atoms are circled in light blue and yellow, respectively. $Ti_3C_2$ sheets stack in a zig-zag structure with respect to bilayers Au.

`

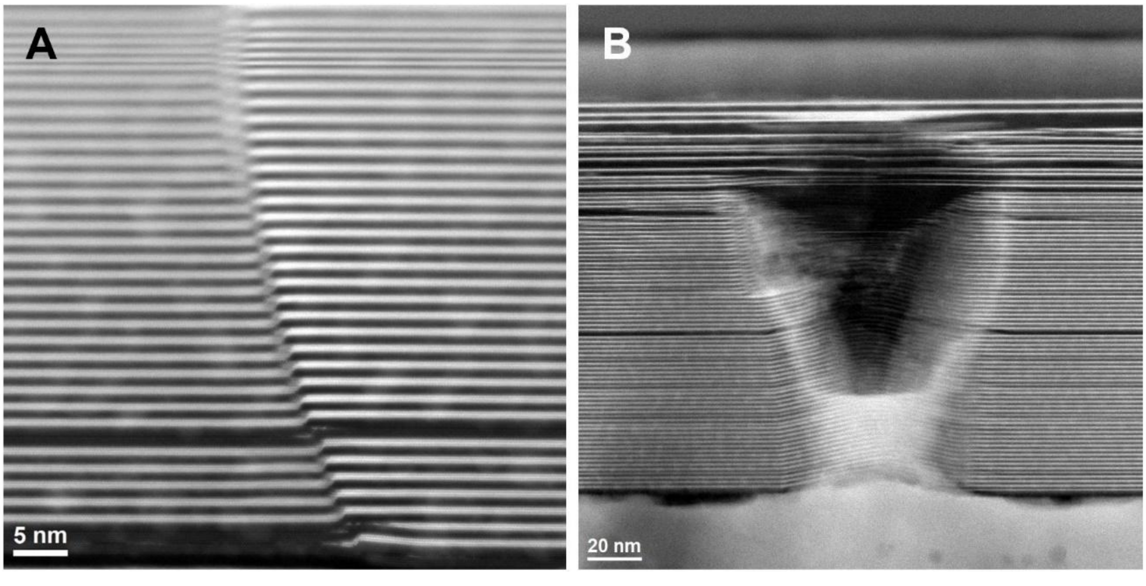


**Fig. S17. Dislocations and ripplocations in $Ti_4Au_3C_3$.**

## Section S6: Preparation of trilayer goldene

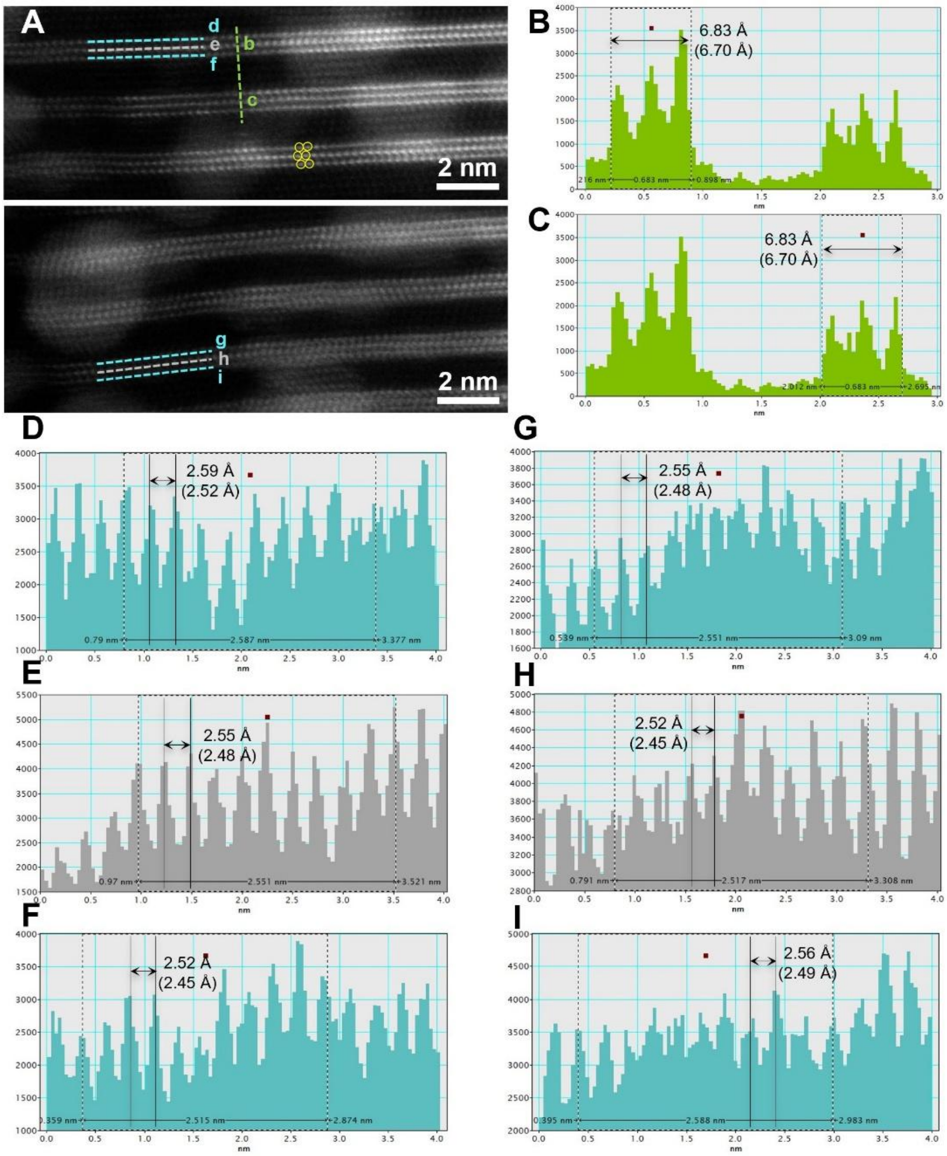


**Fig. S18. In-plane Au-Au spacings and the thickness of trilayer goldene.** (**A**) Cross-sectional HRSTEM image of the trilayer goldene after etching. Au atoms are circled in yellow. (**B** and **C**) The measured thickness of trilayer goldene is around 6.70 Å on average. The distances were calibrated using the reference *c*-parameter of $Ti_4Au_3C_3$ (32.30 Å). (**D** to **I**) Line profiles extracted from the dash lines highlighted in (A). Values in parentheses are calibrated distances.

**Table S5. Average distances obtained from line profiles in Fig. S18 and calculated in-plane Au-Au distances.**

| Au layers in $Ti_4Au_3C_3$ | *Calibrated average distances from line profiles (Å) | Calculated in-plane Au-Au distances (Å) |
|---|---|---|
| Both Sides | 2.49 | 2.88 |
| Middle | 2.47 | 2.85 |

*The distance of two crests in line profiles is equal to √3/2 times the in-plane Au-Au distance.

**Fig. S19** shows blobs and thicker layers formation of Au at the edges of a $Ti_4Au_3C_3$ film after etching. Free gold atoms in the solution would be adsorbed on goldene surfaces with many dangling bonds. These Au adatoms tends to coalesce with surface atoms and form 3D blobs and thicker goldene layers.

Coalescence of Au is also driven by the ion milling during TEM sample preparation (**Fig. S20**). When the ion beam bombards the sample, some of the free gold atoms that are milled away from the film can be re-absorbed and precipitate on the surface of tri-layers Au. Thus, blobs are formed at the edge of a $Ti_4Au_3C_3$ film (close to the milling hole) before etching.

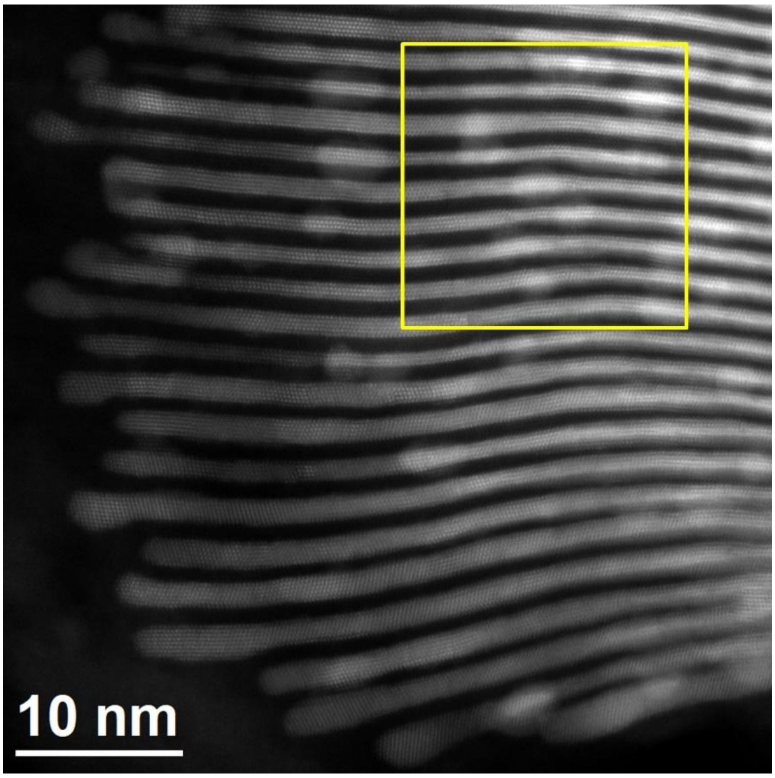


**Fig. S19. Blobs and thicker layers formation of Au at the edges of a $Ti_4Au_3C_3$ film after etching.** Cross-sectional HRSTEM image of trilayer goldene obtained from $Ti_4Au_3C_3$ MAX phase by etching with 0.5 % Murakami's reagent with 5 mM of CTAB for 168 h. Squared region is magnified and displayed in **Fig. 3C**.

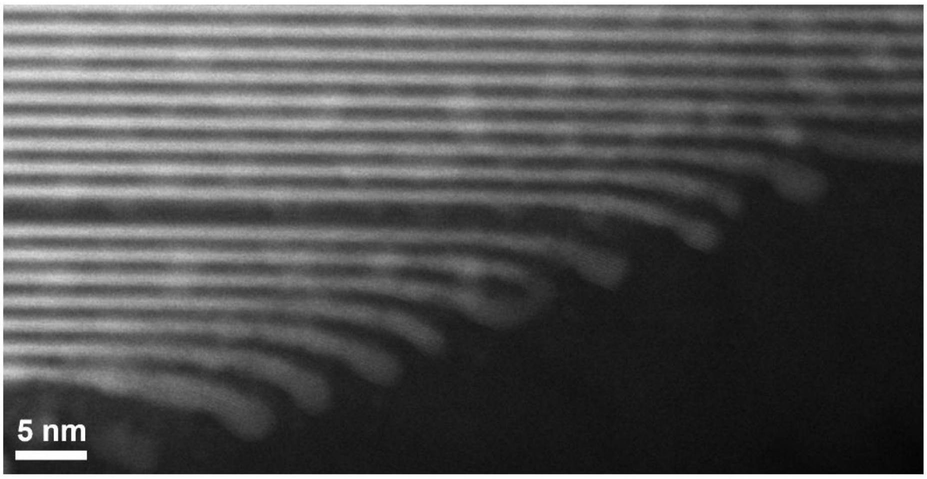


**Fig. S20. Blob formation at the edges of a $Ti_4Au_3C_3$ film.** Cross-sectional HRSTEM image of an unetched $Ti_4AuC_3$ film after ion milling treatment showing blobs and thicker-layers formation of Au at the edges.

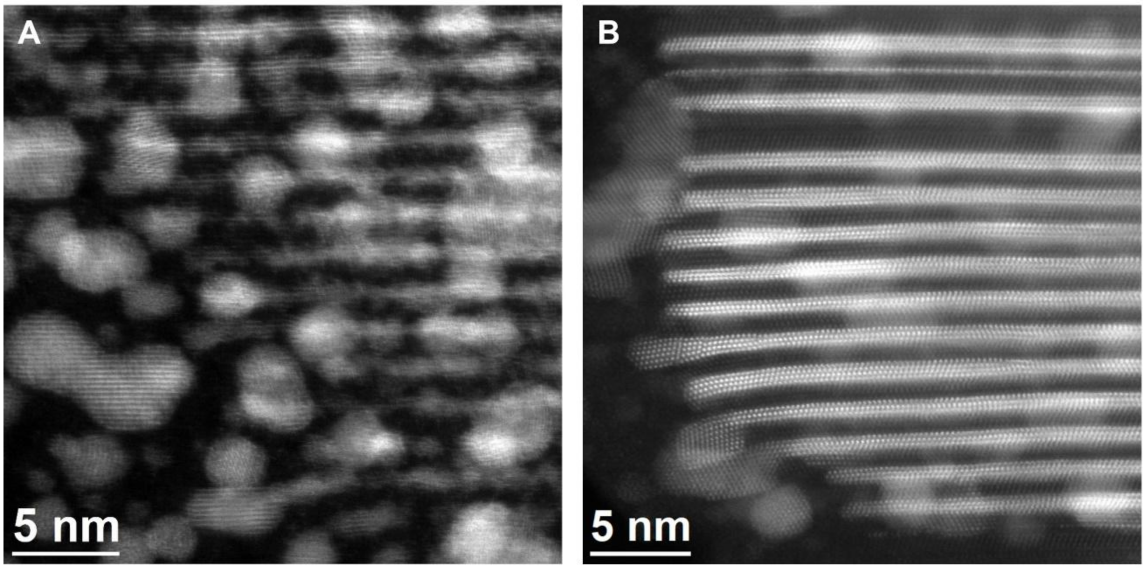


**Fig. S21. Etching $Ti_4Au_3C_3$ films with different etchant concentrations.** Cross-sectional HRSTEM image of $Ti_4Au_3C_3$, after etching by 1 % (**A**) and 0.2 % (**B**) Murakami's reagent with CTAB.

When producing monolayer goldene by etching $Ti_3AuC_2$ without surfactants, we have observed that Au spherical clusters and nanoparticles formed. This verifies the essential role of surfactants in stabilizing goldene sheets by the present and likely other methods.

For producing trilayer goldene, we recently supplemented such a comparison experiment, that is etching the $Ti_4Au_3C_3$ film using the same concentration of etchant (0.5% Murakami) without surfactants. The STEM image after etching is shown in **Fig. S22**. The trilayer Au retains its original structure and does not decompose to form clusters after being released from $Ti_4Au_3C_3$. The magnified image shows that the trilayer goldene becomes rippled, but it is still stable in the absence of CTAB as a stabilizer. This also proves the intrinsic stability of the trilayer goldene and verifies our DFT and AIMD calculation results.

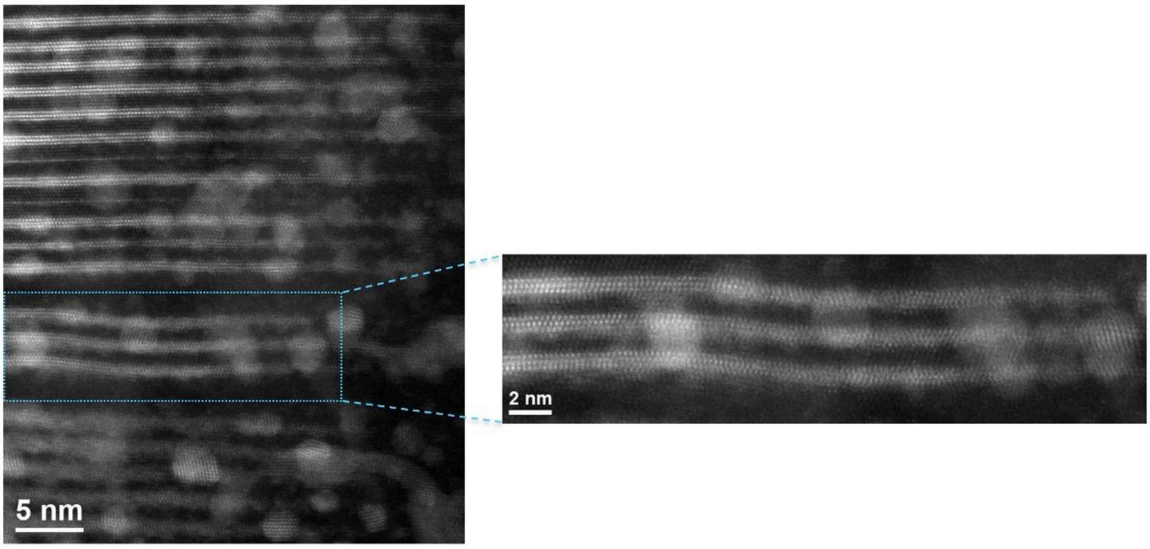


**Fig. S22. Trilayer goldene prepared from etching $Ti_4Au_3C_3$ without surfactants.** Cross-sectional HRSTEM image of $Ti_4Au_3C_3$ after etching by 0.5 % Murakami's reagent without CTAB. The magnified region shows some isolated trilayer goldene flakes with rippled features but stable in the absence of stabilizers.

However, we still believe that the addition of surfactants is beneficial to the dispersion of goldene flakes and prevents them from curling and wrinkling. As previously reported (*68*), characteristic $CH_2$ and $CH_3$ vibration peaks were observed in FT-IR spectrum, indicating CTAB association with the Au cluster; CTAB molecules specifically adsorbed on the gold clusters might form surface ion pairs with Br- ions attached to the Au surfaces, and the cationic CTAB headgroups surround the $Br^-$ layer by electrostatic interactions.

About the stability of goldene sheets, in our original 'monolayer goldene' work (*1*), goldene was subject to electron beam damage under 300 keV irradiation. Thus, our presented images were snapshots taken after first focusing on an adjacent area. **Fig. S23** shows a STEM image of goldene acquired after beam irradiation for several tens of seconds. A free-standing monolayer goldene, which was initially well defined, became blurred over that time span. Additional irradiation for several minutes resulted in the deformation of the goldene and finally the formation of nanoparticles.

In contrast, trilayer goldene in this work is very stable and not easy to aggregate. As observations in STEM images in the main text and the supplementary information, trilayer goldene sheets can withstand 300 KeV irradiation for several minutes or even longer and can maintain the original structures and positions unchanged, allowing us to acquire high-resolution STEM images and EDX signal.

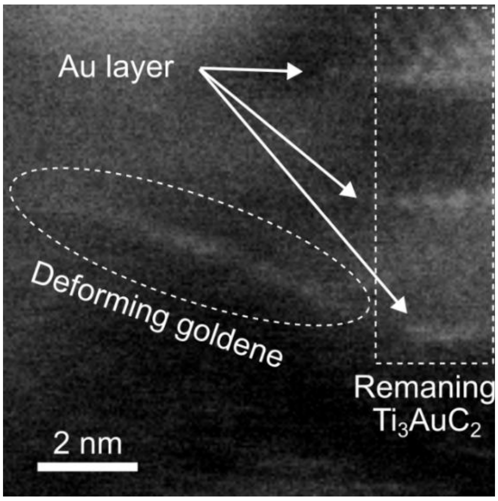


**Fig. S23. Deformation of goldene under 300 keV electron beam exposure.** Cross-sectional HR-STEM image of deforming goldene obtained by etching $Ti_3AuC_2$. Within several minutes of irradiation, the goldene decomposed into spherical Au particle while the $Ti_3AuC_2$ remained.

As for the yield of trilayer goldene preparation, we estimate that a few nanometers of film near the surface was etched away during 7-days etching, generating a few nanolayers of goldene. However, the practical yield would be smaller as most goldene sheets produced are expected to be dispersed in the etchant solution. In terms of surface area, the etching yield is roughly up to 40 % judging from a 30 × 30 $\mu m^2$ PEEM image. This result basically coincides with our original 'monolayer goldene' article (*1*), wherein the XPS Au 4*f* emissions contain goldene (40 %) and gold nanoparticles resulting from the failed etching (60 %), and the plan-view STEM image contains roughly 40% goldene flakes.

## Section S7: DFT and AIMD computations for trilayer goldene

All simulations are performed using the VASP code (*51*) implemented with the projector augmented-wave method (*53*). In present density functional theory **(**DFT) calculations, the electronic exchange and correlation energies are treated in two ways: using the approximation of Perdew-Burke-Ernzerhof (PBE) (*54*) and the local density approximation (LDA). Only the PBE approximation is used in our *ab initio* molecular dynamics (AIMD) simulations. AIMD simulations are assisted by on-the-fly machine-learning (as implemented in VASP.6.4), which allows considerable extension of the simulation time. Spin-orbit effects are neglected in all cases.

Trilayer gold slabs with *ABC* stacking sequence are alike an *fcc* Au crystal surface with {111} termination. Trilayer Au with *ABA* stacking sequence is equivalent to a {0001}- oriented *hcp* lattice surface. DFT calculations are done using 6-atom supercells. The accuracies are of $10^{-6}$ eV/supercell for total energies and $10^{-3}$ eV/Å for forces. Saturation of energy values is achieved with 500 eV cutoff energy for the planewave basis set and 17 × 17 × 1 $\Gamma$-centered *k*-point grids. The supercell area of *fcc* and *hcp* trilayer Au is iteratively adjusted until lateral $|\sigma_{xx}|$ and $|\sigma_{yy}|$ stresses are smaller than 0.1 GPa. A vacuum region of constant thickness (≈ 12 Å) separates slab replicas along the surface normal direction. Thus, the atoms are allowed to relax to minimize supercell energy and interatomic forces. In AIMD simulations, each supercell has 224 atoms per layer (surface areas ≈ 4 × 4 $nm^2$), with 672 Au atoms in total. The simulation cells are periodic in-plane (i.e., within {111} and {0001} planes).

AIMD simulations at 300 K allow us to verify the dynamical stability of *fcc* and *hcp*-stacked trilayer Au slabs and to assess their difference in free-energy. The simulations are carried out using the PBE functional, 1-fs timestep integration, with 300 eV cutoff energy and $\Gamma$-point sampling of the reciprocal space. The NVT ensemble employs the Nose-Hoover thermostat. We construct supercells with in-plane nearest neighbor distance set equal to the one computed by DFT at 0 K. During dynamics, we observe that the lateral stresses $|\sigma_{xx}|$ and $|\sigma_{yy}|$ remain small: ≤0.1 GPa for *fcc* trilayer Au and ≤0.3 GPa for *hcp* trilayer Au. Thanks to the speed-up provided by on-fly-machine-learning implemented in VASP, we could follow the dynamics for 0.18 ns (*fcc* structure) and 0.38 ns (*hcp* structure). ML-assisted AIMD simulations show that the vertical interlayer distance between *fcc*-like slabs increases to 2.680 Å at 300 K (0.75% increase in relation to DFT). For *hcp*-structured trilayer Au, the room-temperature interplanar spacing becomes 2.703 Å (1.54% increase in comparison to the 0 K value).

For both Au allotropes, the in-plane Au-Au nearest neighbor distance obtained by DFT+PBE relaxation is 2.807 Å. DFT+LDA calculations predict 2.745 Å. These values are 4.5% and 4.2% smaller than the corresponding ones computed by DFT+PBE (2.940 Å), and DFT+LDA (2.865 Å) for fcc bulk Au. Experimental measurements yield an interatomic spacing (2.884 Å) that is slightly larger/smaller than predicted.

DFT results show that different stacking sequences have different interlayer spacing. For *fcc*-like trilayer Au, DFT+PBE calculations give an equilibrium interlayer distance of 2.660 Å, while LDA yields a much lower value of 2.517 Å. For *hcp*-like trilayer Au, the relaxed distances between {0001} lattice planes are 2.662 Å (PBE) and 2.524 Å (LDA).

Besides the time-averaged potential energies, the vibrational free energy $F_{vib}$ also contributes to stabilization of trilayer goldene. The Helmholtz free energy ($F$) of the two goldene allotropes is calculated by adding $F_{vib}$ to the time-averaged potential energies. As implemented in Ref. (*69*), the $F_{vib}$ is computed by integrating the vibrational density of states (VDOS). The latter is obtained by Fourier-transforming the velocity-velocity autocorrelation function, which is directly extracted from MD trajectories.

**Fig. S24** shows that the stabilizing contribution of lattice vibrations in *hcp* trilayer goldene is largely due to the low-frequency VDOS peak centered at ≈ 0.3 THz. Note that low-frequency anharmonic vibrations give stronger contribution to vibrational free energy.

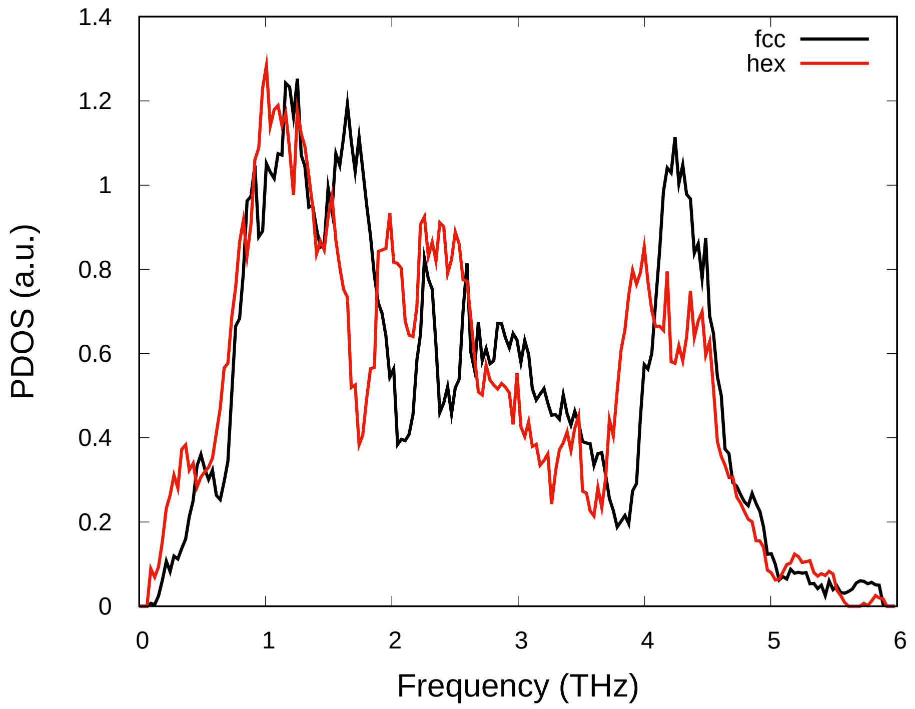


**Fig. S24. VDOS spectra of *fcc* and *hcp* trilayer goldene.**

Time-averaged (±1 ps) atomic positions of *fcc* and *hcp* trilayer goldene were extracted during AIMD trajectories at room temperature, as shown in **Fig. S25**. The atomic positions are consistent with *fcc* (**Fig. S25A** to **C**) and *hcp* (**Fig. S25D** to **F**) stacking sequences. The Helmholtz free energies ($F$) calculated for the two allotropes indicate that the *hcp* trilayer goldene is ≈ 50 meV/atom more stable than the *fcc* trilayer goldene at 300 K. Therefore, we predict that the *hcp* phase would be retained after etching and its stability would contribute to the prevalence of trilayer goldene.

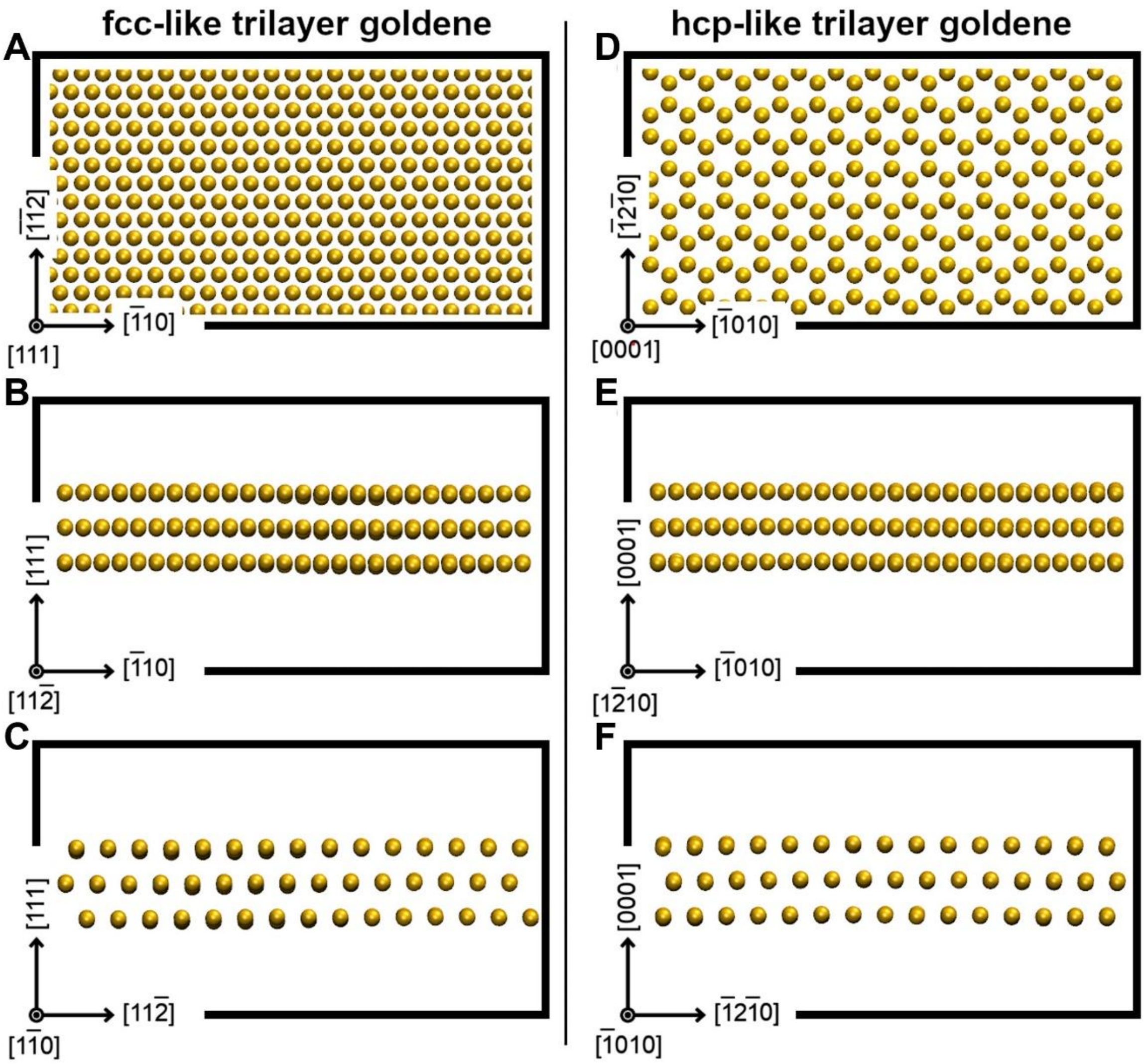


**Fig. S25. Dynamic stability of *fcc* and *hcp* trilayer goldene.** Time-averaged (±1 Picoseconds) atomic positions of *fcc* (**A** to **C**) and *hcp* (**D** to **F**) trilayer goldene sheets recorded from different projections.

## Section S8: Calculated XPS core-level shifts in DFT

Electronic structures were calculated within the density-functional-theory framework and PAW methods. Isolated trilayer and monolayer goldene were calculated using 29×29×3 *k*-points, while bulk Au was calculated with 29×29×29 *k*-points sampling of the Brillouin zone using a Monkhorst-Pack scheme (*70*). The energy cut-off for plane waves included in the expansion of wave functions was 400 eV. The Au $4f_{7/2}$ core level shifts of isolated trilayer and monolayer of goldene with respect to the bulk Au were calculated (**Table S6**) according to the slater transition state, initial state, and final state approximation (*71-73*). The Slater Transition State of bulk Au was used as a reference to obtain the calculated binding energies without accounting for the 4*f* spin-orbit splitting of 3.6 eV. The half-occupied Slater Transition State has previously been proven useful for metallic systems (*74*, *75*).

Applying the initial state approximation, the Au $4f_{7/2}$ core level of monolayer goldene exhibits a low-energy shift compared to that of bulk Au due to the lack of charge transfer between the Au atoms in goldene, while the difference between the trends using both initial and final state approximations are small, implying that final-state effects should also be small (*76*). On the other hand, considering the initial state approximation, the calculated Au $4f_{7/2}$ core level shift of isolated monolayer goldene is toward lower binding energy compared to that of bulk, while that of goldene for the final state effect is smaller than the initial state approximation by 0.376 eV, indicating there is a final state effect for goldene. The lower binding energy using the initial state approximation for goldene in comparison to bulk Au can be attributed to the 2D nature of goldene and lattice distortion (*77*). The reduced coordination number of Au atoms for anisotropic Au nanostructures such as goldene accompanies corresponding charge redistribution, leading to fewer 5*d* electrons of Au atoms than for bulk Au. Furthermore, a lattice distortion with shorter Au-Au distances in goldene than in bulk Au could also cause a subsequent rehybridization that enhances the initial state effect.

The calculations were made assuming infinite free-standing 2D planes of goldene with 30 Å vacuum on both sides. For comparison, the size of the trilayer goldene sheets observed in the STEM measurements varies from several nm to a hundred nm. Such nanosheets have a much higher edge-to-volume ratio than the infinite 2D structure. The coordination number at the edges is lower than 9, enhancing the final state effect. Therefore, the goldene nanosheets produced by our experimental method show a slightly higher positive binding energy shift of 0.80 eV with respect to the bulk Au than estimated in the calculations.

**Table S6. DFT-simulated Au 4$f_{7/2}$ core level shifts of bulk *fcc* Au, trilayer Au (middle and surface layers) and goldene monolayer using three different methods.**

| Sample | Slater transition state approx. [eV] | Initial state approx. [eV] | Final state approx. (incl. initial state approx.) [eV] |
|---|---|---|---|
| Bulk Au | (90.03) | - | - |
| Trilayer mid | +0.018 (90.04) | -0.011 (90.01) | +0.016 (90.04) |
| Trilayer surf | -0.314 (89.71) | -0.422 (89.60) | -0.272 (89.75) |
| Monolayer goldene | -0.452 (89.57) | -0.645 (89.38) | -0.269 (89.76) |

## Section S9: Surface morphology of $Ti_4Au_3C_3$ after etching

**Fig. S26** shows the SEM images and corresponding EDX spectra and maps measured on the $Ti_4Au_3C_3$ surface after chemical-mechanical polishing (CMP) of Au capping layer and selective etching of $Ti_4C_3$ slabs. Both dark and bright regions in panel A exhibit relatively low content of Au (panel B and D to H), indicating the Au capping layer is highly removed after CMP and chemical etching. However, it may partially remain on top of $Ti_4Au_3C_3$. The dark domains are Si-rich because Si out-diffuses into Au reservoir and agglomerates near the surface during Au intercalation processes.

After etching, the surface is full of rippling flakes (panel C), which are most likely the trilayer goldene floating in different orientations. When the $Ti_4Au_3C_3$ film is immersed in the solution, the etchant starts to penetrate into the film from the surface and etch the top few atomic $Ti_4C_3$ slabs gradually. Meanwhile, the released goldene sheets begin rippling due to the activity of surfactant and the tension of the liquid. The rippling feature illustrates that the trilayer goldene completely separates from the support of the formwork and can maintain its original structure. The tendency of goldene sheets curling-up and clustering can be expected.

Therefore, we attribute the less intense high-energy Au 4*f* doublet in the XPS spectrum from etched $Ti_4Au_3C_3$ (**Fig. 4**, main text) to the final state effects due to the decreasing coordination number at the sheet edges of nascent trilayer goldene and conclude that the dominant 4*f* doublet may originate from residuals of the capping Au layer or from sheets clustering and their curling-up.

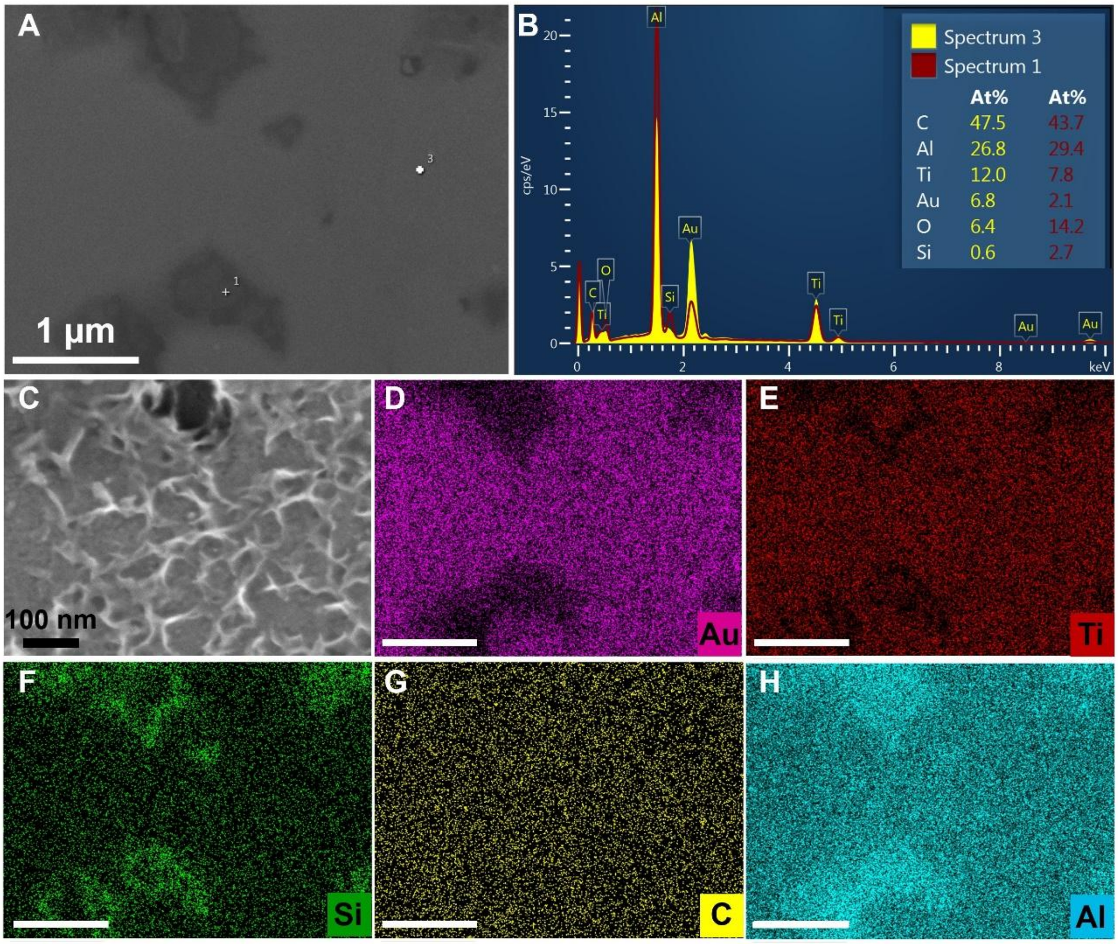


**Fig. S26. SEM image with EDX analysis measured on $Ti_4Au_3C_3$ after etching.** (**A**) Plan view SEM electron image of $Ti_4Au_3C_3$ after etching. (**B**) EDX spectra acquired at two points in (A). (**C**) Magnified plan view SEM image of $Ti_4Au_3C_3$ after etching. (**D** to **H**) EDX maps taken together with (A).

## Section S10: XPS analysis of elements in samples from etching reagents

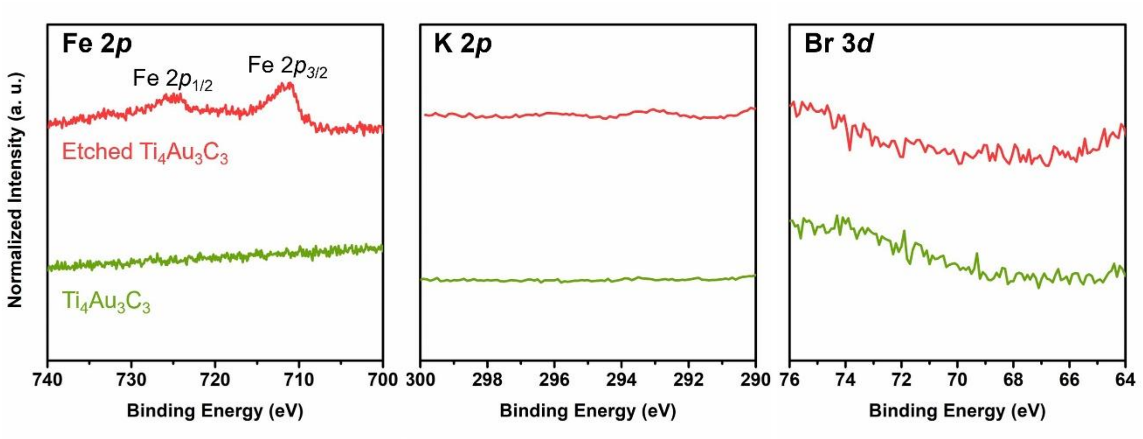


**Fig. S27. X-ray photoelectron spectra of the etched $Ti_4Au_3C_3$ and pristine $Ti_4Au_3C_3$.** Fe 2*p* (left), K 2*f* (middle) and Br 3*d* (right) core level spectra measured on the $Ti_4Au_3C_3$ (green) and trilayer goldene (red) produced from $Ti_4Au_3C_3$ via chemical etching.

All XPS spectra were charge-referenced to the Fermi edge recorded from the etched $Ti_4Au_3C_3$ and pristine $Ti_4Au_3C_3$ samples (**Fig. S28**). The exact position of the Fermi edge was determined by taking the spectra derivative in the vicinity of the Fermi level. The such-obtained peaks were shifted to lower binding energy by 0.1 and 0.15 eV for $Ti_4Au_3C_3$ and etched $Ti_4Au_3C_3$, respectively. Accordingly, the core level XPS spectra were also shifted towards lower binding energy by corresponding amounts.

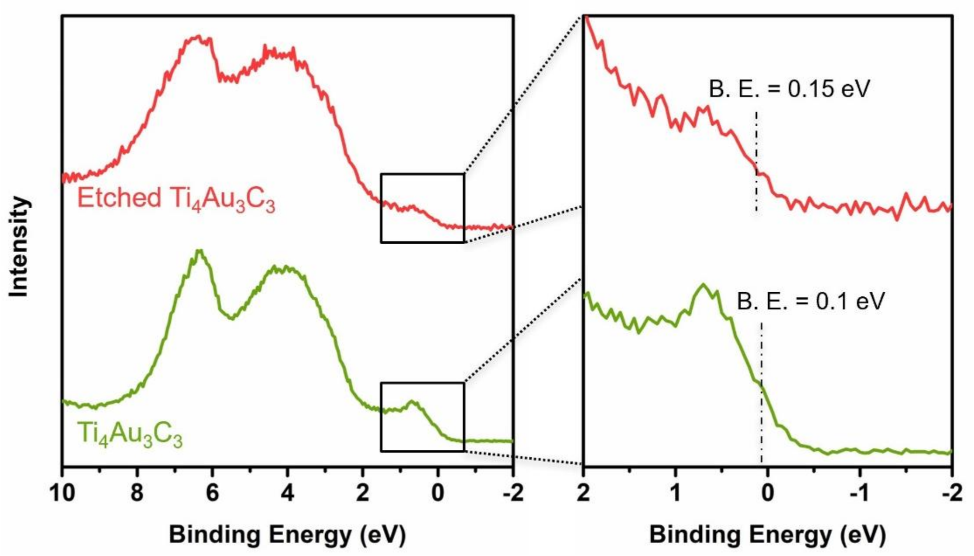


**Fig. S28. Valence band XPS spectra of the etched $Ti_4Au_3C_3$ and pristine $Ti_4Au_3C_3$.**

## Section S11: Goldene layering effects

The relationship between conductivity and the density of states (DOS) at the Fermi level ($D(E_F)$) in gold changes as the number of monolayers increases due to quantum confinement effects and the transition from a 2D (thin film) system to a 3D (bulk) metal.

In ultra-thin gold films (a few monolayers), the electronic states are confined in the direction perpendicular to the film. This quantization confinement modifies the density of states. $D(E_F)$ becomes reduced or non-uniform in such thin films because the continuous energy bands of bulk gold are replaced by discrete energy levels. This reduction in $D(E_F)$ decreases the number of available conduction electrons, leading to lower conductivity compared to the bulk material. In thin films, conduction electrons experience enhanced scattering at surfaces and interfaces, reducing the relaxation time ($\tau$). Surface scattering contributes substantially to the reduction in conductivity for thin films. As the number of monolayers increases, the confinement effects weaken, and $D(E_F)$ approaches the bulk value. Surface scattering diminishes as the bulk-like structure emerges, leading to a longer relaxation time ($\tau$). When the thickness becomes comparable to or larger than the electron mean free path, the conductivity becomes closer to that of bulk gold.

The conductivity ($\sigma$) is generally given by: $\sigma = e^2 D(E_F)(V_F)^2\tau$, where $D(E_F)$, the Fermi velocity ($V_F$), and the relaxation time ($\tau$) are all affected by the number of monolayers. For few monolayers, $D(E_F)$ is reduced due to quantum confinement, and $\tau$ is smaller due to surface scattering, leading to lower conductivity.

For many monolayers, $D(E_F)$ increases and approaches the bulk value, while $\tau$ also increases as bulk scattering dominates over surface scattering. Conductivity rises accordingly. As the number of monolayers in gold increases, the DOS at the Fermi level ($D(E_F)$) transitions from a confined, reduced state to the bulk value. Gradually increases, eventually approaching the bulk conductivity of gold as the number of monolayers increases and quantum confinement effects diminish.

In addition, a recent study (*20*) suggests that ‘the conductance of goldene monolayer is equal to that of a gold film with a thickness of about 1 nm. And the undulations of the actual structure of any goldene sample are inevitable, which had been observed in the original study of synthesizing the goldene sample. The structural undulation, also called ripples, is ubiquitous in many 2D materials of single-atomic-layer thickness, e.g., goldene and graphene. As a kind of disorder, undulations will impact necessarily the electronic transport property in goldene to some extent.’ From this perspective, the undulation of trilayer goldene in the normal direction

of the sample sheets should be weaker than that of monolayer goldene, thereby reducing the scattering of electrons in transport process and improving conductivity.

Therefore, our consideration is that the conductivity of monolayer and trilayer should be very close, but of trilayer would be slightly higher.

## REFERENCES AND NOTES


1. S. Kashiwaya, Y. Shi, J. Lu, D. G. Sangiovanni, G. Greczynski, M. Magnuson, M. Andersson, J. Rosén, L. Hultman, Synthesis of goldene comprising single-atom layer gold. *Nat. Synth.* **3**, 744–751 (2024).

2. M. Xu, T. Liang, M. Shi, H. Chen, Graphene-like two-dimensional materials. *Chem. Rev.* **113**, 3766–3798 (2013).

3. Y. Chen, Z. Fan, Z. Zhang, W. Niu, C. Li, N. Yang, B. Chen, H. Zhang, Two-dimensional metal nanomaterials: Synthesis, properties, and applications. *Chem. Rev.* **118**, 6409–6455 (2018).

4. N. L. Rosi, D. A. Giljohann, C. S. Thaxton, A. K. R. Lytton-Jean, M. S. Han, C. A. Mirkin, Oligonucleotide-modified gold nanoparticles for intracellular gene regulation. *Science* **312**, 1027–1030 (2006).

5. D. Astruc, F. Lu, J. R. Aranzaes, Nanoparticles as recyclable catalysts: The frontier between homogeneous and heterogeneous catalysis. *Angew. Chem. Int. Ed. Engl.* **44**, 7852–7872 (2005).

6. M. Hu, J. Chen, Z.-Y. Li, L. Au, G. V. Hartland, X. Li, M. Marquez, Y. Xia, Gold nanostructures: Engineering their plasmonic properties for biomedical applications. *Chem. Soc. Rev.* **35**, 1084–1094 (2006).

7. S. Bhandari, B. Hao, K. Waters, C. H. Lee, J.-C. Idrobo, D. Zhang, R. Pandey, Y. K. Yap, Two-dimensional gold quantum dots with tunable bandgaps. *ACS Nano* **13**, 4347–4353 (2019).

8. S. Forti, S. Link, A. Stöhr, Y. Niu, A. A. Zakharov, C. Coletti, U. Starke, Semiconductor to metal transition in two-dimensional gold and its van der Waals heterostack with graphene. *Nat. Commun.* **11**, 2236 (2020).

9. S. Ye, A. P. Brown, A. C. Stammers, N. H. Thomson, J. Wen, L. Roach, R. J. Bushby, P. L. Coletta, K. Critchley, S. D. Connell, A. F. Markham, R. Brydson, Sub-nanometer thick gold nanosheets as highly efficient catalysts. *Adv. Sci.* **6**, 1900911 (2019).

10. M. Herran, S. Juergensen, M. Kessens, D. Hoeing, A. Köppen, A. Sousa-Castillo, W. J. Parak, H. Lange, S. Reich, F. Schulz, E. Cortés, Plasmonic bimetallic two-dimensional supercrystals for $H_2$ generation. *Nat. Catal.* **6**, 1205–1214 (2023).

11. L. Yang, P. Wang, Z. Yang, Y. Pei, Effect of thiolate-ligand passivation on the electronic structure and optical absorption properties of ultrathin one and two-dimensional gold nanocrystals. *Nanoscale* **9**, 5554–5566 (2020).

12. L. Wang, Y. Zhu, J. Q. Wang, F. Liu, J. Huang, X. Meng, J.-M. Basset, Y. Han, F. S. Xiao, Two-dimensional gold nanostructures with high activity for selective oxidation of carbon-hydrogen bonds. *Nat. Commun.* **6**, 6957 (2015).

13. X. Wang, C. Wang, C. Chen, H. Duan, K. Du, Free-standing monatomic thick two-dimensional gold. *Nano Lett.* **19**, 4560–4566 (2019).

14. J. Fox, G. Newham, R. J. Bushby, E. M. A. Valleley, P. L. Coletta, S. D. Evans, Spectrophotometric analysis and optimization of 2D gold nanosheet formation. *J. Phys. Chem. C* **127**, 3067–3076 (2023).

15. L. Zhao, H. Q. Ta, R. G. Mendes, A. Bachmatiuk, M. H. Rummeli, In situ observations of freestanding single-atom-thick gold nanoribbons suspended in graphene. *Adv. Mater. Interfaces* **7**, 2000436 (2020).

16. W. Yuan, Z. Deng, Z. Ren, Y. Shen, W. Xi, J. Luo, Monolayer goldene intercalated in graphene layers. *Appl. Phys. Lett.* **117**, 233102 (2020).

17. S. Chahal, A. Bandyopadhyay, S. P. Dash, P. Kumar, Microwave synthesized 2D gold and its 2D-2D hybrids. *J. Phys. Chem. Lett.* **13**, 6487–6495 (2022).

18. L.-M. Yang, M. Dornfeld, T. Frauenheim, E. Ganz, Glitter in a 2D monolayer. *Phys. Chem. Chem. Phys.* **17**, 26036–26042 (2015).

19. L.-M. Yang, A. B. Ganz, M. Dornfeld, E. Ganz, Computational study of quasi-2D liquid state in free standing platinum, silver, gold, and copper monolayers. *Condens. Matter* **1**, 1 (2016).

20. S. Zhao, H. Zhang, M. Zhu, L. Jiang, Y. Zheng, Electrical conductivity of goldene. *Phys. Rev. B* **110**, 085111 (2024).

21. M. Dahlqvist, M. W. Barsoum, J. Rosén, MAX phases—Past, present, and future. *Mater. Today* **72**, 1–24 (2024).

22. A. VahidMohammadi, J. Rosen, Y. Gogotsi, The world of two-dimensional carbides and nitrides (MXenes). *Science* **372**, eabf1581 (2021).

23. M. Naguib, M. Kurtoglu, V. Presser, J. Lu, J. Niu, M. Heon, L. Hultman, Y. Gogotsi, M. W. Barsoum, Two-dimensional nanocrystals produced by exfoliation of $Ti_3AlC_2$. *Adv. Mater.* **23**, 4248–4253 (2011).

24. M. S. Mironov, D. I. Yakubovsky, G. A. Ermolaev, I. A. Khramtsov, R. V. Kirtaev, A. S. Slavich, G. I. Tselikov, A. A. Vyshnevyy, A. V. Arsenin, V. S. Volkov, K. S. Novoselov, Graphene-inspired wafer-scale ultrathin gold films. *Nano Lett.* **24**, 16270–16275 (2024).

25. H. Fashandi, M. Dahlqvist, J. Lu, J. Palisaitis, S. I. Simak, I. A. Abrikosov, J. Rosen, L. Hultman, M. Andersson, A. L. Spetz, P. Eklund, Synthesis of $Ti_3AuC_2$, $Ti_3Au_2C_2$ and $Ti_3IrC_2$ by noble metal substitution reaction in $Ti_3SiC_2$ for high-temperature-stable Ohmic contacts to SiC. *Nat. Mater.* **16**, 814–818 (2017).

26. H. Okamoto, T. B. Massalski, The Au−Si (Gold-Silicon) system. *Bull. Alloy Phase Diagr.* **4**, 190–198 (1983).

27. H. Fashandi, C.-C. Lai, M. Dahlqvist, J. Lu, J. Rosen, L. Hultman, G. Greczynski, M. Andersson, A. L. Spetz, P. Eklund, $Ti_2Au_2C$ and $Ti_3Au_2C_2$ formed by solid state reaction of gold with $Ti_2AlC$ and $Ti_3AlC_2$. *Chem. Commun.* **53**, 9554–9557 (2017).

28. S. Wan, X. Li, Y. Chen, N. Liu, Y. Du, S. Dou, L. Jiang, Q. Cheng, High-strength scalable MXene films through bridging-induced densification. *Science* **374**, 96–99 (2021).

29. S. Peng, A. C. Meng, M. R. Braun, A. F. Marshall, P. C. McIntyre, Plasmons and inter-band transitions of hexagonal close packed gold nanoparticles. *Appl. Phys. Lett.* **115**, 051107 (2019).

30. I. Chakraborty, S. N. Shirodkar, S. Gohil, U. V. Waghmare, P. Ayyub, A stable, quasi-2D modification of silver: Optical, electronic, vibrational and mechanical properties, and first principles calculations. *J. Phys. Condens. Matter* **26**, 025402 (2014).

31. X. Huang, S. Li, Y. Huang, S. Wu, X. Zhou, S. Li, C. L. Gan, F. Boey, C. A. Mirkin, H. Zhang, Synthesis of hexagonal close-packed gold nanostructures. *Nat. Commun.* **2**, 292 (2011).

32. Z. Fan, M. Bosman, X. Huang, D. Huang, Y. Yu, K. P. Ong, Y. A. Akimov, L. Wu, B. Li, J. Wu, Y. Huang, Q. Liu, C. E. Png, C. L. Gan, P. Yang, H. Zhang, Stabilization of 4H hexagonal phase in gold nanoribbons. *Nat. Commun.* **6**, 7684 (2015).

33. Y. Kondo, K. Takayanagi, Gold nanobridge stabilized by surface structure. *Phys. Rev. Lett.* **79**, 3455–3458 (1997).

34. H. Cheng, N. Yang, Q. Lu, Z. Zhang, H. Zhang, Syntheses and properties of metal nanomaterials with novel crystal phases. *Adv. Mater.* **30**, 1707189 (2018).

35. S. Hu, X. Wang, Ultrathin nanostructures: Smaller size with new phenomena. *Chem. Soc. Rev.* **42**, 5577–5594 (2013).

36. X. Huang, H. Li, S. Li, S. Wu, F. Boey, J. Ma, H. Zhang, Synthesis of gold square-like plates from ultrathin gold square sheets: The evolution of structure phase and shape. *Angew. Chem. Int. Ed. Engl.* **50**, 12245–12248 (2011).

37. E. C. Groesbeck, Metallographic etching reagents. *Sci. Pap. Bur. Stand.* **20**, 518 (1925).

38. T. K. Sau, C. J. Murphy, Room temperature, high-yield synthesis of multiple shapes of gold nanoparticles in aqueous solution. *J. Am. Chem. Soc.* **126**, 8648–8649 (2004).

39. M. Grzelczak, J. Pérez-Juste, P. Mulvaney, L. M. Liz-Marzán, Shape control in gold nanoparticle synthesis. *Colloidal Synthesis of Plasmonic Nanometals* 197–220 (2020).

40. D. K. Smith, B. A. Korgel, The importance of the CTAB surfactant on the colloidal seed-mediated synthesis of gold nanorods. *Langmuir* **24**, 644–649 (2008).

41. Z. Fan, H. Zhang, Crystal phase-controlled synthesis, properties and applications of noble metal nanomaterials. *Chem. Soc. Rev.* **45**, 63–82 (2016).

42. International Organization for Standardization, *Surface chemical analysis—X-ray photoelectron spectrometers—Calibration of energy scales*. ISO 15472:2010 (ISO, 2020).

43. M. Magnuson, O. Wilhelmsson, J.-P. Palmquist, U. Jansson, M. Mattesini, S. Li, R. Ahuja, O. Eriksson, Electronic structure and chemical bonding in $Ti_2AlC$ investigated by soft x-ray emission spectroscopy. *Phys. Rev. B* **74**, 195108 (2006).

44. L.-Å. Näslund, P. O. Å. Persson, J. Rosén, X-ray photoelectron spectroscopy of $Ti_3AlC_2$, $Ti_3C_2T_z$, and TiC provides evidence for the electrostatic interaction between laminated layers in MAX-phase materials. *J. Phy. Chem. C* **124**, 27732–27742 (2020).

45. G. K. Wertheim, S. B. DiCenzo, S. E. Youngquist, Unit charge on supported gold clusters in photoemission final state. *Phys. Rev. Lett.* **51**, 2310–2313 (1983).

46. S. B. DiCenzo, S. D. Berry, E. H. Hartford Jr., Photoelectron spectroscopy of single-size Au clusters collected on a substrate. *Phys. Rev. B* **38**, 8465–8468 (1988).

47. S. Peters, S. Peredkov, M. Neeb, W. Eberhardt, M. Al-Hada, Size-dependent XPS spectra of small supported Au-clusters. *Sur. Sci.* **608**, 129–134 (2013).

48. J. F. Moulder, W. F. Stickle, P. E. Sobol, K. D. Bomben, Handbook of X-ray photoelectron spectroscopy. *Phys. Electron. Inc.* 261 (1992).

49. Y. Shi, S. Kashiwaya, P. Helmer, J. Lu, M. Andersson, A. Petruhins, J. Rosén, L. Hultman, Synthesis of $Cr_2AuC$ via thermal substitution reaction in Au-covered $Cr_2GaC$ and $Cr_2GeC$ thin films. *Results Mater.* **18**, 100403 (2023).

50. K. N. Tu, Selective growth of metal-rich silicide of near-noble metals. *Appl. Phys. Lett.* **27**, 221–224 (1975).

51. G. Kresse, J. Furthmuller, Efficient iterative schemes for ab initio total-energy calculations using a plane-wave basis set. *Phys. Rev. B* **54**, 11169–11186 (1996).

52. G. Kresse, D. Joubert, From ultrasoft pseudopotentials to the projector augmented-wave method. *Phys. Rev. B* **59**, 1758–1775 (1999).

53. P. E. Blochl, Projector augmented-wave method. *Phys. Rev. B* **50**, 17953–17979 (1994).

54. J. P. Perdew, K. Burke, M. Ernzerhof, Generalized gradient approximation made simple. *Phys. Rev. Lett.* **77**, 3865–3868 (1996).

55. A. Jain, S. P. Ong, G. Hautier, W. Chen, W. D. Richards, S. Dacek, S. Cholia, D. Gunter, D. Skinner, G. Ceder, K. A. Persson, Commentary: The Materials Project: A materials genome approach to accelerating materials innovation. *APL Mater.* **1**, 011002 (2013).

56. M. Dahlqvist, B. Alling, I. A. Abrikosov, J. Rosén, Phase stability of $Ti_2AlC$ upon oxygen incorporation: A first-principles investigation. *Phys. Rev. B* **81**, 024111 (2010).

57. M. Dahlqvist, B. Alling, J. Rosén, Stability trends of *MAX* phases from first principles. *Phys. Rev. B* **81**, 220102 (2010).

58. R. Dronskowski, P. E. Bloechl, Crystal orbital Hamilton populations (COHP): Energy-resolved visualization of chemical bonding in solids based on density-functional calculations. *J. Phys. Chem.* **97**, 8617–8624 (1993).

59. V. L. Deringer, A. L. Tchougreeff, R. Dronskowski, Crystal orbital Hamilton population (COHP) analysis as projected from plane-wave basis sets. *J. Phys. Chem. A* **115**, 5461–5466 (2011).

60. S. Maintz, V. L. Deringer, A. L. Tchougreeff, R. Dronskowski, Analytic projection from plane-wave and PAW wavefunctions and application to chemical-bonding analysis in solids. *J. Comput. Chem.* **34**, 2557–2567 (2013).

61. K. Momma, F. Izumi, *VESTA 3* for three-dimensional visualization of crystal, volumetric and morphology data. *J. Appl. Crystallogr.* **44**, 1272–1276 (2011).

62. G. Henkelman, A. Arnaldsson, H. Jónsson, A fast and robust algorithm for Bader decomposition of charge density. *Comput. Mater. Sci.* **36**, 354–360 (2006).

63. Q. Zhu, G. Cao, J. Wang, C. Deng, J. Li, Z. Zhang, S. X. Mao, In situ atomistic observation of disconnection-mediated grain boundary migration. *Nat. Commun.* **10**, 156 (2019).

64. M. F. Ashby, Boundary defects, and atomic aspects of boundary sliding and diffusional creep. *Sur. Sci.* **31**, 498–542 (1972).

65. M. W. Barsoum, Ripplocations: A progress report. *Front. Mater.* **7**, 146 (2020).

66. M. W. Barsoum, T. Zhen, S. R. Kalidindi, M. Radovic, A. Murugaiah, Fully reversible, dislocation-based compressive deformation of $Ti_3SiC_2$ to 1 GPa. *Nat. Mater.* **2**, 107–111 (2003).

67. Q. Zhu, L. Kong, H. Lu, Q. Huang, Y. Chen, Y. Liu, W. Yang, Z. Zhang, F. Sansoz, H. Zhou, J. Wang, Revealing extreme twin-boundary shear deformability in metalic nanocrystals. *Sci. Adv.* **7**, eabe4758 (2021).

68. W. Cheng, S. Dong, E. Wang, Synthesis and self-assembly of cetyltrimethylammonium bromide capped gold nanoparticles. *Langmuir* **19**, 9434–9439 (2003).

69. O. V. Pshyk, X. Li, I. Petrov, D. G. Sangiovanni, J. Palisaitis, L. Hultman, G. Greczynski, Discovery of Guinier-Preston zone hardening in refractory nitride ceramics. *Acta Mater.* **255**, 119105 (2023).

70. H. J. Monkhorst, J. D. Pack, Special points for Brillouin-zone integrations. *Phys. Rev. B* **13**, 5188–5192 (1976).

71. L. Köhler, G. Kresse, Density functional study of CO on Rh(111). *Phys. Rev. B* **70**, 165405 (2004).

72. S. Lizzit, A. Baraldi, A. Groso, K. Reuter, M. V. Ganduglia-Pirovano, C. Stampfl, M. Scheffler, M. Stichler, C. Keller, W. Wurth, D. Menzel, Surface core-level shifts of clean and oxygen-covered Ru(0001). *Phys. Rev. B* **63**, 205419 (2001).

73. A. A. Tal, W. Olovsson, I. A. Abrikosov, Origin of the core-level binding energy shifts in Au nanoclusters. *Phys. Rev. B* **95**, 245402 (2017).

74. C. Göransson, W. Olovsson, I. A. Abrikosov, Numerical investigation of the validity of the Slater-Janak transition-state model in metallic systems. *Phys. Rev. B* **72**, 134203 (2005).

75. W. Olovsson, T. Marten, E. Holmström, B. Johansson, I. A. Abrikosov, First principle calculations of core-level binding energy and Auger kinetic energy shifts in metallic solids. *J. Elec. Spec. Rel. Phen.* **178-179**, 88–99 (2010).

76. M. Weinert, R. E. Watson, Core-level shifts in bulk alloys and surface adlayers. *Phys. Rev. B* **51**, 17168–17180 (1995).

77. E. Costanzo, G. Faraci, A. R. Pennisi, S. Ravesi, A. Terrasi, G. Margaritondo, Initial and final state effects in photoemission from gold clusters. *Solid State Commun.* **81**, 155–158 (1992).
